# Development of (4-Phenylamino)quinazoline Alkylthiourea Derivatives as Novel NF-κB Inhibitors

**DOI:** 10.3390/ph15070778

**Published:** 2022-06-22

**Authors:** Sarah S. Darwish, Po-Jen Chen, Mostafa M. Hamed, Reem A. Wagdy, Shun-Hua Chen, Ashraf H. Abadi, Mohammad Abdel-Halim, Tsong-Long Hwang, Matthias Engel

**Affiliations:** 1 Department of Pharmaceutical Chemistry, Faculty of Pharmacy and Biotechnology, German University in Cairo, Cairo 11835, Egypt; s.sameh@gaf.edu.eg (S.S.D.); reem.ahmed-afifi@guc.edu.eg (R.A.W.); ashraf.abadi@guc.edu.eg (A.H.A.); mohammad.abdel-halim@guc.edu.eg (M.A.-H.); 2 School of Life and Medical Sciences, University of Hertfordshire Hosted by Global Academic Foundation, New Administrative Capital, Cairo 11578, Egypt; 3 Department of Medical Research, E-Da Hospital, Kaohsiung 824005, Taiwan; litlep@hotmail.com; 4 Drug Design and Optimization, Helmholtz Institute for Pharmaceutical Research Saarland (HIPS), Helmholtz Centre for Infection Research, Saarland University Campus, 66123 Saarbrücken, Germany; mostafa.hamed@helmholtz-hips.de; 5 School of Nursing, Fooyin University, Kaohsiung 831, Taiwan; ft104@fy.edu.tw; 6 Graduate Institute of Natural Products, College of Medicine, Chang Gung University, Taoyuan 333, Taiwan; 7 Research Center for Chinese Herbal Medicine, Graduate Institute of Health Industry Technology, College of Human Ecology, Chang Gung University of Science and Technology, Taoyuan 333, Taiwan; 8 Department of Anesthesiology, Chang Gung Memorial Hospital, Taoyuan 333, Taiwan; 9 Department of Chemical Engineering, Ming Chi University of Technology, New Taipei City 243, Taiwan; 10 Pharmaceutical and Medicinal Chemistry, Saarland University, Campus C2.3, 66123 Saarbrücken, Germany

**Keywords:** 4-aminoquinazolines, NF-κB inhibitor, inflammation, macrophage targeting, TNFα, IL-6

## Abstract

For many inflammatory diseases, new effective drugs with fewer side effects are needed. While it appears promising to target the activation of the central pro-inflammatory transcription factor NF-κB, many previously discovered agents suffered from cytotoxicity. In this study, new alkylthiourea quinazoline derivatives were developed that selectively inhibit the activation of NF-κB in macrophage-like THP−1 cells while showing low general cytotoxicity. One of the best compounds, **19**, strongly inhibited the production of IL-6 (IC_50_ = 0.84 µM) and, less potently, of TNFα (IC_50_ = 4.0 µM); in comparison, the reference compound, caffeic acid phenethyl ester (CAPE), showed IC_50_s of 1.1 and 11.4 µM, respectively. Interestingly, **19** was found to block the translocation of the NF-κB dimer to the nucleus, although its release from the IκB complex was unaffected. Furthermore, **19** suppressed the phosphorylation of NF-κB-p65 at Ser468 but not at Ser536; however, **19** did not inhibit any kinase involved in NF-κB activation. The only partial suppression of p65 phosphorylation might be associated with fewer side effects. Since several compounds selectively induced cell death in activated macrophage-like THP−1 cells, they might be particularly effective in various inflammatory diseases that are exacerbated by excess activated macrophages, such as arteriosclerosis and autoimmune diseases.

## 1. Introduction

Chronic inflammatory and autoimmune diseases are characterized by dysregulated production of pro-inflammatory cytokines and macrophage infiltration, which might contribute to detrimental tissue remodeling and destruction [1,2,3,4]. The commonly prescribed anti-inflammatory drugs have potentially serious side effects: NSAIDs are known for their potential to induce gastrointestinal ulceration and bleeding [5]. Many antirheumatic drugs, such as methotrexate, sulfasalazine, and hydroxychloroquine, may cause an increased risk of infection and hepatotoxicity [6,7]. Anti-inflammatory glucocorticoids are effective, but the therapy has metabolic and cardiovascular side effects, and long-term treatment is limited due to the development of resistance [8,9]. Antibodies blocking the major pro-inflammatory cytokine TNFα can cause new-onset neurologic symptoms in rheumatoid arthritis patients, associated with demyelinating lesions of the CNS [10,11], and may elicit immunogenic responses [12]. Moreover, a significant proportion of rheumatoid arthritis patients and up to 40% of the patients with inflammatory bowel diseases fail to achieve a long-term clinical response [13,14].

**Figure 1:**
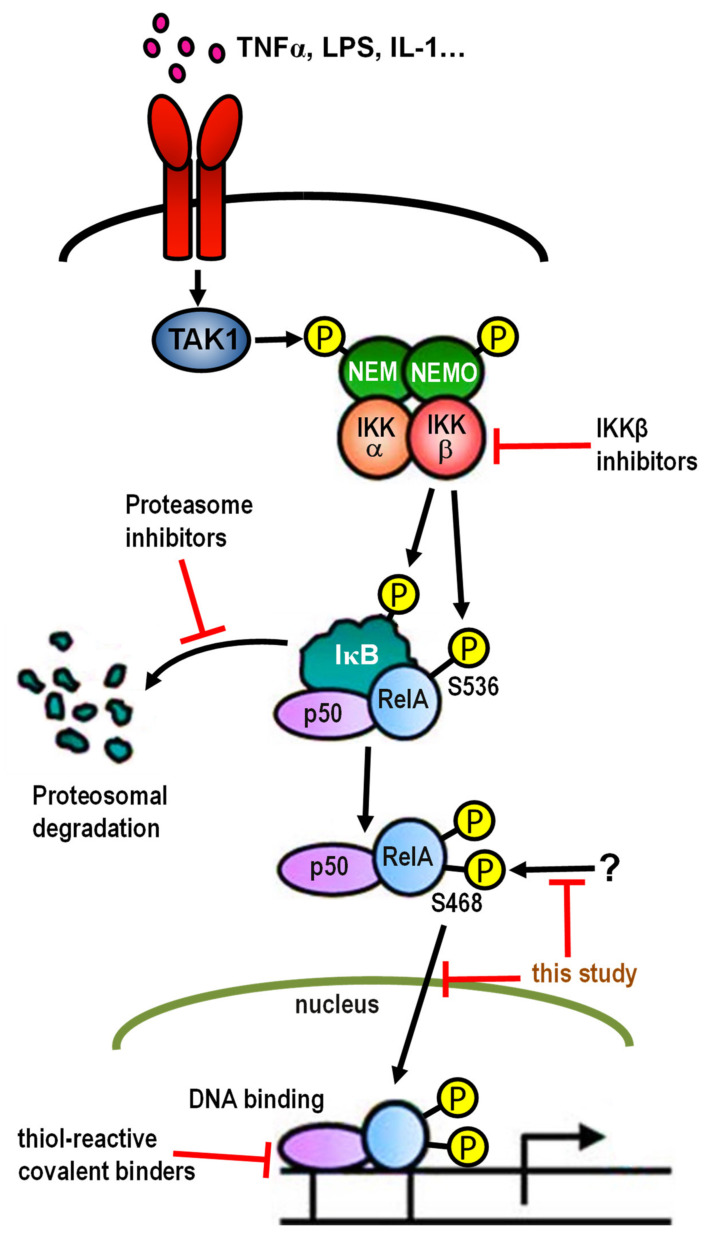
The canonical pathway of NF-κB activation with the most common points of pharmacological intervention. The pathway is activated by signals from various immune-related receptors. In stimulated pro-inflammatory cells, ligand–receptor interactions initially activate the kinase TAK1. Subsequently, TAK1 activates the IKK complex by phosphorylation, leading to IκBα phosphorylation and ubiquitin-dependent proteasomal degradation. Usually, the released NF-κB dimer is rapidly translocated to the nucleus, where it initiates the expression of specific genes; however, phosphorylations on Ser536, Ser468, and other sites on the RelA subunit further regulate stability, DNA-binding activity, and nuclear import. Some classes of inhibitors that block distinct steps in this pathway are indicated by red bars.

Instead of targeting TNFα and other single cytokines, recent drug development approaches aimed at the inhibition of the central pro-inflammatory transcription factor NF-κB, which would result in the dampening of the inflammatory response as a whole (see Figure 1 for illustration). Most of these approaches focused on the inhibition of the upstream activator kinases IKKα/β, leading to the prevention of IκB phosphorylation, which normally keeps the inhibitory complex with the NF-κB dimer intact (Figure 1). Several ATP-competitive and allosteric IKKα/β inhibitors have been described in the last few decades, including 2-amino-3,5-diarylbenzamides [15], imidazo [1,2-a]quinoxaline derivatives [16,17], 4-Phenyl-7-azaindoles [18], and thiazolidine-2,4-diones [19,20]. The best compound, **8h** (from Ref. [15]), was highly selective for the IKKα/β kinases in a screening panel of 150 kinases and displayed a cell free potency against IKKβ of 100 nM, while the translocation of NF-κB to the nucleus was blocked at 2 µM. However, the selectivity of IKKβ, the actual activator of NF-κB release, over the closest homologue, IKKα, was only four-fold. In this respect, the earlier-discovered imidazo [1,2-*a*]quinoxaline BMS-345541 offered some advantage as it was more selective for IKKβ over IKKα, with cell-free IC_50_s of 0.3 and 4 µM, respectively. It was proposed to bind to a yet-unidentified allosteric site on the catalytic subunit. The IC_50_s for the suppression of IL-1β and TNFα production in the THP−1 macrophage model cell line were in the range of 5 µM. Notably, BMS-345541 was also effective in the inhibition of TNFα in mice after administration of LPS. The 4-Phenyl-7-azaindoles were among the most potent IKKβ inhibitors, with compound **16** from the work of Liddle et al. displaying an IC_50_ of 40 nM, an excellent selectivity over IKKα, and, further, 36 screened kinases [18]. In a cellular reporter gene assay, **16** inhibited the NF-κB activation with an IC_50_ of 0.8 µM [18]. Elkamhawy and co-workers reported thiazolidine-2,4-dione-based IKKβ inhibitors with an irreversible, allosteric mode of action [19,20]. The best compounds, **6v** [21] and **7a** [20], inhibited IKKβ in the cell free assay with IC_50_ values of 0.4 and 0.2 µM, respectively, while the potencies to suppress the TNFα production in rat RAW 264.7 macrophage-like cells were 1.7 and 6.3 µM, respectively. Further, **7a** was also active in an in vivo sepsis model in mice [20]. However, the inhibition of IKKβ leads to severe on-target toxicities, including potential tumor-promoting effects, thus lacking clinical success so far (reviewed in Ref. [22]).

Numerous natural compounds from terrestrial and marine organisms were reported to inhibit the activation of NF-κB by a variety of mechanisms [23]; if known at all, these comprise targeting IKKα/β (e.g., cacospongiolide B, IC_50_ = 0.26 µM [24]), inhibition of the activity of the 26S proteasome (e.g., salinosporamide A, IC_50_ = 1.3 nM [25]), and interfering with the binding of NF-κB to its DNA recognition site through covalent modification of the p65 subunit (e.g., helenalin, IC50 = 5 µM [26]) (cf. Figure 1).

Many of these compounds lack drug-likeness and raise concerns with respect to cytotoxicity as they originated as chemical defense mechanisms using cytotoxic secondary metabolites [27]. Some natural compounds known to exhibit pleiotropic activities were reported to also inhibit NF-κB, e.g., curcumin [28,29] and caffeic acid phenethyl ester (CAPE) [30]. CAPE inhibited the activation of NF-κB in cells with an IC_50_ between 10 and 20 µM, for which the Michael reaction acceptor and the catechol motif in the structure were required [31].

An inhibition of the proteosome, which also affects the degradation of IκBα [18] (Figure 1), besides many other protein targets, is exerted by several FDA-approved anti-cancer drugs; however, this strategy induces severe adverse effects, which even concerns the second-generation proteasome inhibitors, such as carfilzomib and ixazomib [32,33,34,35]. In general, many inhibitors of the NF-κB signaling failed in the clinic because of adverse toxic effects [36].

**Figure 2:**
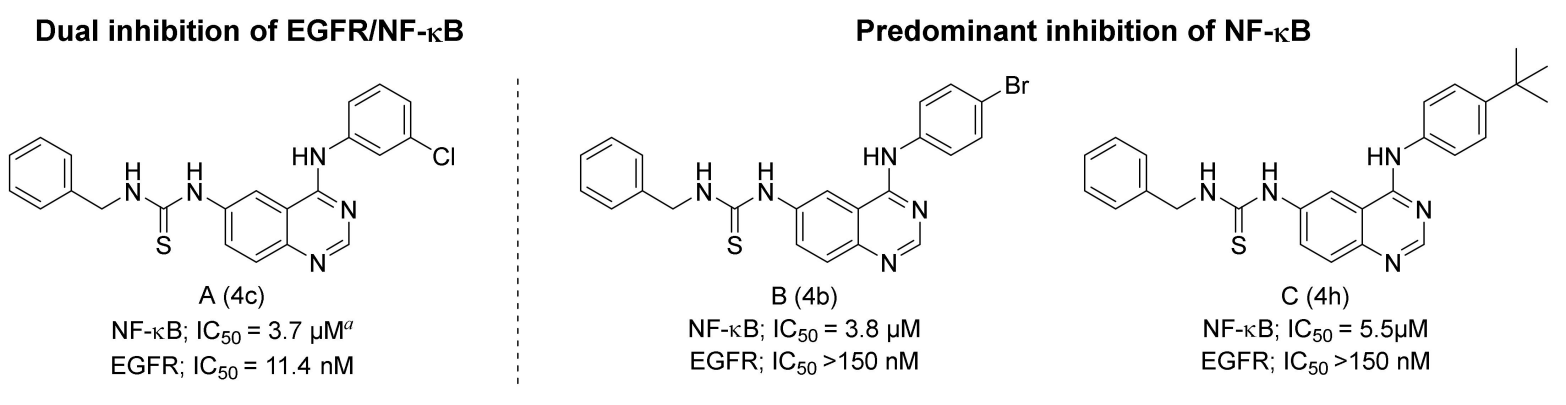
Thiourea quinazoline derivatives from our previous series [35]. *^a^* NF-κB inhibition values were derived from a cell-based activity reporter assay, whereas the IC_50_s for EGFR kinase inhibition were obtained in a cell-free kinase assay (at 100 µM ATP). The codes in brackets denote the old compound names from Ref. [35].

We previously reported a series of benzylthiourea quinazoline derivatives as the first bispecific EGFR/NF-κB inhibitors, which exhibited anti-cancer activity in vitro and in vivo (Figure 2, compound **A**). Basically, these compounds might potentially also be evaluated as anti-inflammatory compounds; however, EGFR kinase inhibition does not contribute to the treatment of inflammatory diseases; rather, it is associated with known on-target side effects, such as cutaneous toxicity [33]. In keratinocytes, EGFR kinase inhibitors cause the induction of growth arrest and apoptosis, which eventually stimulate inflammation [34]. Hence, a potential anti-inflammatory agent should avoid the inhibition of EGFR kinase [35]. Of note, some of the previously published benzylthiourea quinazoline derivatives (compounds **B** and **C**, Figure 2) showed a tendency for predominant inhibition of NF-κB, suggesting that it might be possible to further enhance this activity while reducing the binding affinity to EGFR kinase. In the present study, we optimized the scaffold using a diversification strategy, resulting in novel NF-κB inhibitors with largely reduced EGFR kinase inhibitory activity and low general cytotoxicity. A biological evaluation in macrophage-like THP−1 cells revealed great potential as anti-inflammatory agents.

## 2. Results and Discussion

### 2.1. Compound Design

**Scheme 1:**
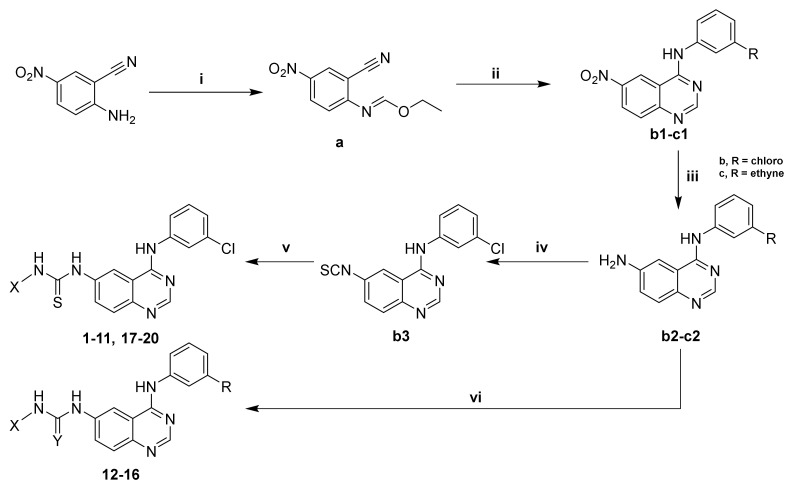
Reagents and conditions: (i) TEOF, (Ac)_2_O, reflux, 16 h; (ii) R-NH_2_, CH_3_COOH, reflux, 1 h; (iii) SnCl_2_, MeOH, reflux, 30 min; (iv) S=CCl_2_, HCl, 3 h; (v) X-NH_2_, DMF, rt, 5 h; (vi) X-NCS or X-NCO, DMF, rt, 5 h.



Our goal was to reduce the binding affinity of the benzylthiourea quinazoline derivatives to the ATP binding pocket of EGFR kinase while retaining the best NF-κB inhibitory activities of the dual inhibitors in the IC_50_ range from 0.3 to 0.7 µM [37]. Although the introduction of a bulky substituent in the 4-position of the aminophenyl group in the published compounds **B** and **C** had reduced the inhibition of EGFR kinase compared with **A**, further modifications at this position did not seem promising because the potency against NF-κB did not increase in parallel but rather dropped with the bulkiness of the substituent in **C**. Therefore, we decided to explore whether modification at the opposite molecule end could also lead to reduced affinity against EGFR kinase, hopefully without impairing the NF-κB inhibition. To this end, we investigated the potential binding mode of the previously published compound **A** in EGFR kinase using molecular docking to evaluate whether substitutions at the benzyl ring might impede binding to the ATP pocket. In addition, the benzyl ring was virtually replaced by a bulky *t*-butyl group to generate a probe compound, whose binding to EGFR kinase was also analyzed by molecular docking (this virtual compound was synthesized later as compound **20**, Scheme 1). Such alkyl thiourea derivatives might offer several advantages compared with substituted benzyl derivatives: a potentially lower molecular weight and overall lipophilicity, as well as a reduced aromatic ring count, which might improve the drug-likeness.

**Figure 3:**
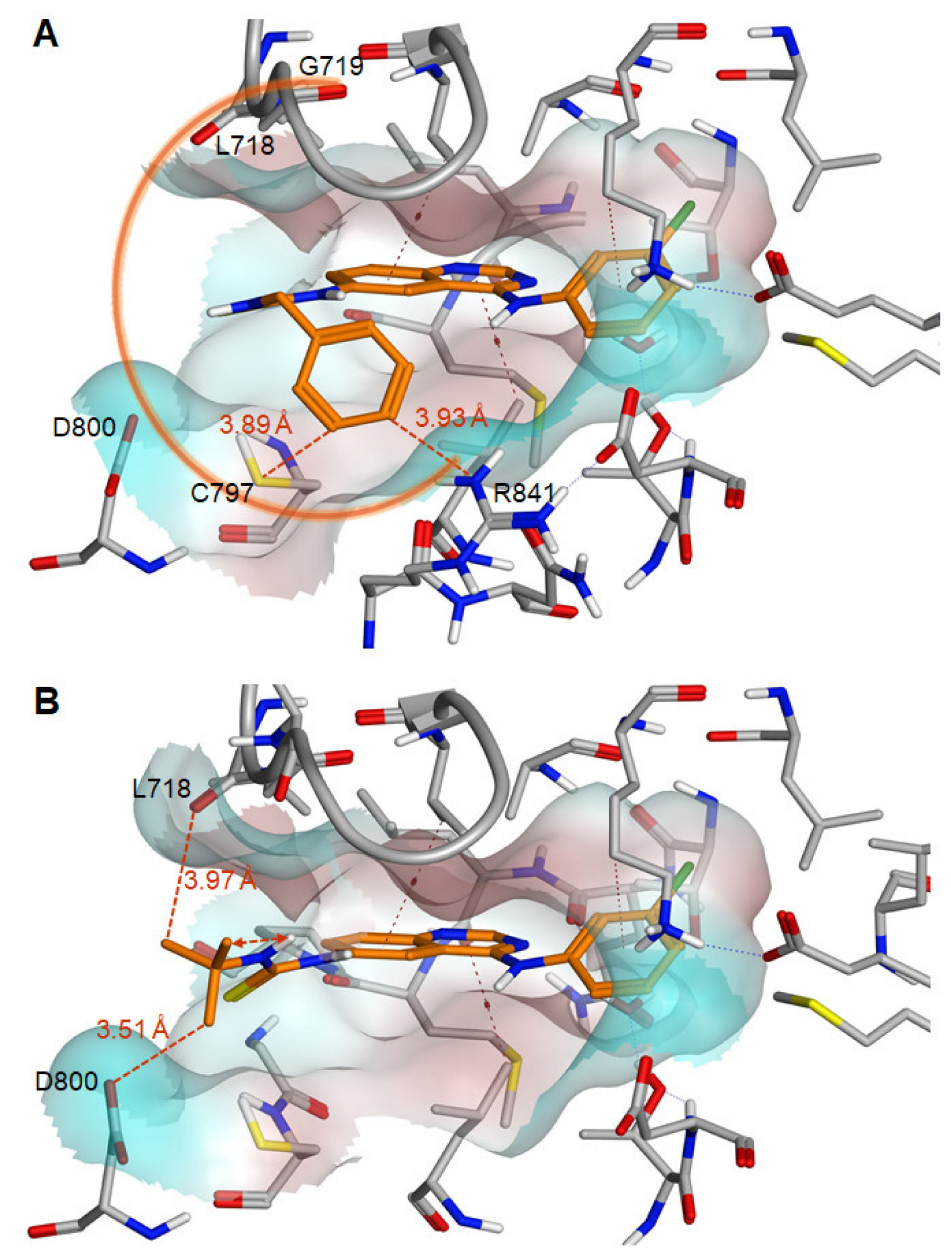
Design concept to abolish the EGFR inhibitory activity based on the predicted binding modes of compounds **A** (**A**) and **20** (**B**) in the ATP pocket of EGFR kinase. Molecular docking was performed to the coordinates of the gefitinib–EGFR cocrystal structure (PDB code: 4WKQ) as previously described using MOE [36,37]. The ATP binding pocket is shown as a transparent Connolly surface with color-encoded lipophilic (brown) and hydrophilic areas (cyan), and the ligands are drawn as orange sticks. The binding mode of the 4-aminophenyl quinazoline core superimposed well with the corresponding portion of gefitinib in the original cocrystal structure (overlay not shown for clarity). In all poses obtained, the benzyl ring in **A** and the *t*-butyl group in **20** were positioned in unfavorable close distances to polar residues at the pocket rim; these distances are shown as orange dashed lines in the depicted representative poses, and the residues are indicated. In other poses, the rotamers of the benzyl group in **A** closely approached the carboxylate of Asp800 or the carbonyls of Leu 718 or Gly719. The radius covered by the benzyl group is illustrated by the orange semicircle. The unfavorable eclipsed conformation of **20** (**B**) is indicated by the double arrow. The glycine-rich loop backbone is represented as a cord. Brown dashed lines: CH–π interactions; blue dashed lines: H-bonds.

The docking results with compound **A** showed that the benzyl ring could bind at several positions of the pocket rim but was always in close distance (≤4 Å) to hydrophilic moieties, consisting either of polar side chains and/or of carbonyl groups from the glycine–rich loop. A representative binding pose is depicted in Figure 3A. The quinazoline ring system and the 4-aminophenyl superimposed well with the corresponding moieties of gefinitib in the original co-crystal structure (overlay not shown). In this pose, the benzyl group was predicted to be within a close distance to Cys797 and Arg841 (3.89 and 3.93 Å, respectively), while, in other poses, enabled by the rotational flexibility of the benzyl group, the carbonyl groups of Leu718 or Gly719 were approached (indicated by the orange semicircle). Hence, we surmised that substituents at the phenyl ring would diminish the overall binding affinity by a steric collision with one or more of the named residues. To this end, we planned to introduce a variety of non-polar substituents since polar moieties had been found to be detrimental to the NF-κB inhibitory activity in our previous study [35].

When the *t*-butyl-modified probe compound (later named **20**) was docked into the EGFR kinase ATP pocket, the increased bulkiness at the thiourea group inevitably caused even more close distances to the pocket residues, as shown in Figure 3B. With the high-affinity interactions of the 4-phenylamino quinazoline core being retained, the *t*-butyl moiety was forced to bind in an unfavorable hydrophilic environment formed by the carbonyl of Leu718 and the Asp800 carboxylate (distances: 3.97 Å and 3.51 Å, respectively). It should be noted that these distances were already maximized at the expense of an energetically unfavorable eclipsed conformation between a *t*-butyl methyl group and the thiourea hydrogen. Altogether, the alkyl modification strategy seemed very promising with respect to the abolishment of EGFR kinase inhibition.

### 2.2. Chemistry

For the generation of our quinazoline derivatives, we adopted a flexible approach that we previously developed, which would facilitate the derivatization at the last step. We started with the synthesis of the quinazoline nucleus, which was conducted through two steps. The first step involved the formation of formimidate derivative **a** (Scheme 1) by refluxing 2-amino-5-nitrobenzonitrile with triethyl orthoformate (TEOF) in the presence of drops of acetic anhydride (Ac_2_O). This was followed by a cyclization step to yield the quinazoline nucleus, whereby the formimidate derivative **a** was refluxed in acetic acid with substituted anilines to yield the nitroquinazoline derivatives **b1**–**c1**. The reduction of the nitroquinazoline derivatives was completed by refluxing with stannous chloride (SnCl_2_) in methanol under a nitrogen atmosphere to yield the aminoquinazoline derivatives **b2**–**c2**. The reduction step was clearly confirmed by the appearance of a signal in the proton NMR spectra at 5.62 ppm, corresponding to the two protons of the “NH_2_”. The synthesis of novel thiourea derivatives was achieved through one of two strategies; the first approach (utilized to synthesize compounds **1**–**11** and **17**–**20**) involved reacting the aminoquinazoline with thiophosgene (S=CCl_2_), yielding the isothiocyanate derivative, which was then stirred with the respective amines to produce thiourea derivatives. The rest of the novel compounds (**12**–**16**) were synthesized via directly reacting the aminoquinazoline derivative with the corresponding isocyanate/isothiocyanate in DMF. The formation of the thiourea was confirmed with the appearance of a clear downfield signal for the (C=S) in the carbon NMR spectra at around 181 ppm. Moreover, the methylene (-CH_2_-) bridge available in compounds (**1**–**14**) was observed in the proton NMR spectra as two protons appearing in the range of 4.37–4.95 ppm.

**Cpd.**

**X**

**Cpd.**

**X**

**R**

**Y**

**1**


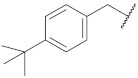


**11**


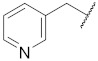

--
**2**


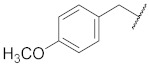


**12**


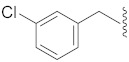

ClO
**3**


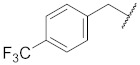


**13**


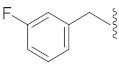

ClO
**4**


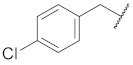


**14**


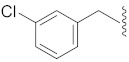



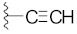

O
**5**


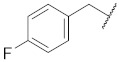


**15**


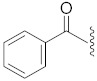

ClO
**6**


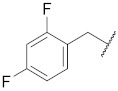


**16**


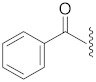

ClS
**7**


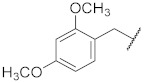


**17**


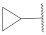

--
**8**


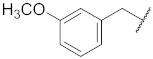


**18**


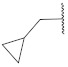

--
**9**


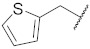


**19**


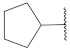

--
**10**


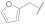


**20**


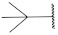

--


### 2.3. Structure–Activity Relationships (SAR)

#### 2.3.1. Benzylthiourea Quinazoline Derivatives

We decided to explore the effect of diverse benzyl substitutions on the NF-κB and EGFR inhibitory activity. In this endeavor, a *meta*-chloro aniline residue was kept constant at the 4-quinazoline site, which ensured a good basal activity for NF-κB inhibition and was not too bulky, thus avoiding strong steric constraints upon binding. Unfortunately, decorating the benzyl moiety with different substituents in varying positions did not sufficiently diminish EGFR kinase inhibition (compounds **1**–**7**, Table 1), suggesting that the substituted benzyl group can adopt binding modes in which the phenyl ring protrudes freely into the solvent so that steric clashes of the substituent(s) with the binding pocket are largely avoided. Although this also implies a loss of the contribution of the benzyl group to the binding, the remaining interactions of the 4-amino quinazoline core still accounted for a rather high binding affinity to the EGFR kinase ATP pocket. Several *para* substitutions of the phenyl ring were detrimental to the activity against NF-κB too (compounds **1**, **3**, **4,** and **7**). Only with compound **8** (Table 1), the EGFR kinase inhibition was reduced to 40% at 150 nM while still displaying significant NF-κB inhibitory activity. Bioisosteric replacement of the benzyl ring did not result in any improvement either (compounds **9**–**11**) (Table 1). Altogether, the benzyl moiety at the thiourea was found unsuitable to enhance the NF-κB suppression over the EGFR kinase inhibition; however, the cytotoxicity remained at a low level, with many NF-κB inhibitory benzyl derivatives (*cf.* **A**, **2**, **6** and **8**) proving that NF-κB suppression by this scaffold and cytotoxicity are not necessarily coupled.

Next, we tried further benzyl modifications, such as benzyl urea and benzoyl (thio)urea motifs (**12**–**16**, Table 2); the rationale behind this was to increase the polarity of the benzyl and the thiourea linker by introducing carbonyl groups, which could lead to repulsion from hydrophobic areas of the EGFR kinase binding site. However, while we could observe a tendency towards weaker inhibition of EGFR kinase (**13** and **15**), the inhibitory activity against NF-κB was affected as well (Table 2).

#### 2.3.2. Alkylthiourea Quinazoline Derivatives

Pursuing our second design strategy, we finally replaced the benzyl group by several alkyl and cycloalkyl moieties (Table 3), also including the *t*-butyl group (**20**) originally tested as a virtual compound (Figure 3B). Indeed, this strategy led to compounds with clearly reduced EGFR kinase inhibition, acceptable NF-κB suppression, and low cytotoxicity (**19**–**20**).

In summary, our design strategy to introduce bulk alkyl groups at the thiourea linker led to several new compounds with greatly reduced effects on EGFR kinase while being low µM inhibitors of NF-κB in the reporter gene assay, in particular **19** and **20**. In the next step, we decided to evaluate the most promising compounds in a more meaningful inflammatory cell model using macrophage-like THP−1 cells. THP−1 is a human leukemia monocytic cell line that can be differentiated to a macrophage-like phenotype using, e.g., phorbol 12-myristate 13-acetate (PMA); these differentiated THP−1 (dTHP−1) cells are an established model to assess the effects of drugs or toxins on the pro-inflammatory macrophage activities by measuring the mRNA expression or release of cytokines [38,39]. Following differentiation, THP−1 cells can be stimulated, e.g., by bacterial lipopolysaccharides (LPS), similar to primary monocyte-derived human macrophages [40].

### 2.4. Evaluation of the Anti-Inflammatory Activities in Macrophage-like dTHP−1 Cells

The most promising compounds from the present study were selected for an evaluation of their macrophage-modulatory activities (Table 4). We chose compounds **19** and **20**, exhibiting the most balanced activity profile in the precedent assays, but also included further compounds, such as **2**, **6**, and **18**, which still displayed moderate inhibition of EGFR kinase in the cell free assay but were optimal in the other criteria, in particular regarding cytotoxicity. Furthermore, we also included **7** and **17**, which were weak inhibitors of the NF-κB activity in the reporter gene assay (IC_50_ > 10 µM), to analyze whether the screening data from the NF-κB reporter assay in HEK293 cells would correlate with the potency to suppress cytokine release from macrophage-like cells.

Following the differentiation of the THP−1 cells by PMA, the compounds were added to the medium and the inflammatory response of the cells was induced by LPS. After 24 h, the levels of TNFα and IL-6 released to the medium were analyzed (Table 4). Somewhat unexpectedly, we observed a strong suppression of IL-6 production by all the compounds, including those that had shown rather poor activity in the NF-κB activity reporter assay (**7** and **17**). Further, **6** was identified as the most potent compound, exhibiting an almost complete blockage of the IL-6 release at the 7.5 µM screening concentration and a sub-micromolar IC_50_ of 0.36 µM. In contrast, it appeared more challenging to inhibit the production of TNFα because it is the predominant cytokine released by dTHP−1 cells and produced earlier and in considerably larger amounts than IL-6 following stimulation by LPS [41], although both IL-6 and TNFα are NF-κB-dependent genes. Hence, it could be expected that a more drastic inhibition of the NF-κB activation pathway would be required to significantly reduce the TNFα levels over 24 h. Our compounds were, accordingly, less potent to inhibit the TNFα production; nevertheless, several compounds achieved single-digit µM potencies, with **6** and **19** being the most potent (IC_50_ = 4.4 and 4.0 µM, respectively), also outperforming the reference compound, CAPE. Interestingly, **17** and **7**, classified as weak inhibitors in the NF-κB activity reporter assay, were also the least potent to suppress the TNFα expression in the dTHP-1 cells. In addition, three of the four compounds selected because of their good activity in the NF-κB reporter assay (**6**, **19,** and **20**) were also the most potent inhibitors of TNFα release. Thus, some correlation could be observed, but it was not stringent, which might be ascribed to the different background of transcriptional co-factors and further protein complex compositions in the NF-κB signaling pathway in HEK293 cells versus vs. dTHP−1 cells.

In parallel, we also analyzed whether our NF-κB inhibitors would affect the viability of the LPS-stimulated dTHP−1 cells (Table 5). In the absence of LPS at the test concentration of 7.5 µM, the compounds exhibited only moderate to low cytotoxicity toward the macrophage-like cells; however, after the activation by LPS, some compounds showed a pronounced cytotoxic effect, particularly **6** and **19**. In contrast, **17** and **7**, which were the weakest NF-κB inhibitors, did not affect the cell viability at 7.5 µM, **7** not even at 15 µM. The reference compound, CAPE, also triggered enhanced cell death in the presence of LPS but to a lower extent, paralleling the less potent inhibition of TNFα production. Thus, it was evident that the strength to selectively kill activated macrophage-like dTHP−1 cells correlated with the potency to inhibit NF-κB, as indicated by the suppression of TNFα release.

How can this selective cytotoxicity be explained? Macrophages are intrinsically resistant to TNFα-induced cell death; however, it was previously shown that, when NF-κB is inhibited in macrophages, TNFα alters the lysosomal membrane permeability, leading to the release of cathepsin B, with the subsequent loss of the inner mitochondrial transmembrane potential (ΔΨm) and cell death [42]. Thus, since the TNFα release is not completely blocked, small amounts of this cytokine in the medium can trigger cell death when the NF-κB pathway is sufficiently inhibited. Since, particularly, **6** and **19** showed only little or no toxicity against HEK293 and A549 cells (Table 1 and Table 3), they might be applicable to selectively deplete activated macrophages in inflammatory disorders, where the presence of a tissue macrophage is generally associated with a poor clinical outcome.

### 2.5. Investigation of the Compounds’ Mode of Action in dTHP−1 Cells

#### 2.5.1. The Alkylthiourea Quinazoline Derivative 19 Inhibits the Translocation of NF-κB to the Nucleus

**Figure 4:**
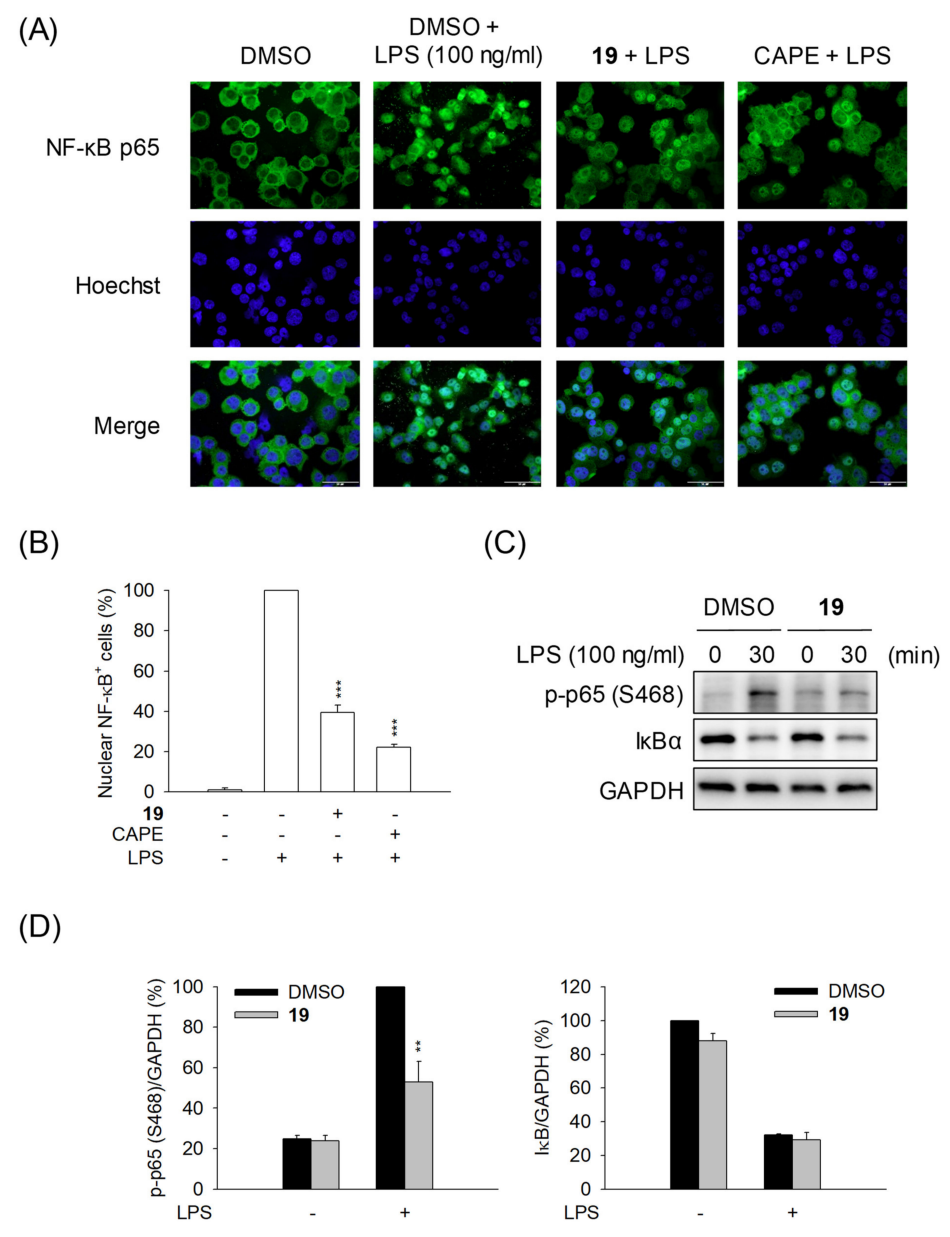
Compound **19** inhibits the LPS-induced nuclear translocation of NF-κB p65 in differentiated THP−1 (dTHP−1) cells. The dTHP−1 cells were incubated with DMSO (0.1%), **19** (7.5 µM), or CAPE (10 µM) in medium for 3 h and then stimulated with or without LPS (0.1 µg/mL) for another 30 min. (**A**) Nuclear translocation of NF-κB p65 (green) was determined by immunofluorescent microscopy. Hoechst (blue) was used for nuclei staining. (**B**) The nuclear NF-κB p65-positive cells were quantified. (**C**) The levels of phospho-NF-κB p65 (S468), IκB, and GAPDH were determined by Western blot. (**D**) The normalized phospho-NF-κB p65 (S468) and IκB levels were quantified. All data are expressed as mean ± SEM (*n* = 3); ** *p* < 0.01 and *** *p* < 0.001 compared with the DMSO + LPS group.

To close in on the point of intervention of our compounds within the NF-κB signaling pathway, we started by analyzing whether one of the best compounds, **19**, inhibits the translocation of the NF-κB dimer (consisting of p65/RelA and p50) to the nucleus. After the stimulation of dTHP−1 cells by LPS, it could be observed that **19** clearly inhibited the nuclear translocation of NF-κB (Figure 4A,B). This result confirmed that the inhibition of cytokine expression was due to the retention of the NF-κB dimer in the cytoplasm, thus preventing the NF-κB-dependent transactivation of the target genes. Up to this point, the mechanism of action of our compounds was indistinguishable from that of the reference compound, CAPE.

#### 2.5.2. Compound **19** Does Not Inhibit IκB Degradation and NF-κB Release

A frequently exploited intervention site targeted by many previous compounds is the IκB kinase (IKK), which phosphorylates the inhibitory protein IκBα, thus triggering its degradation and the release of the NF-κB dimer. A further phosphorylation site of IKK, and of several other kinases too, is Ser536 at p65/RelA (reviewed in Ref. [43]). The phosphorylation of Ser536 may promote the nuclear translocation of NF-κB independent from the regulation by IκBα [44].

**Figure 5:**
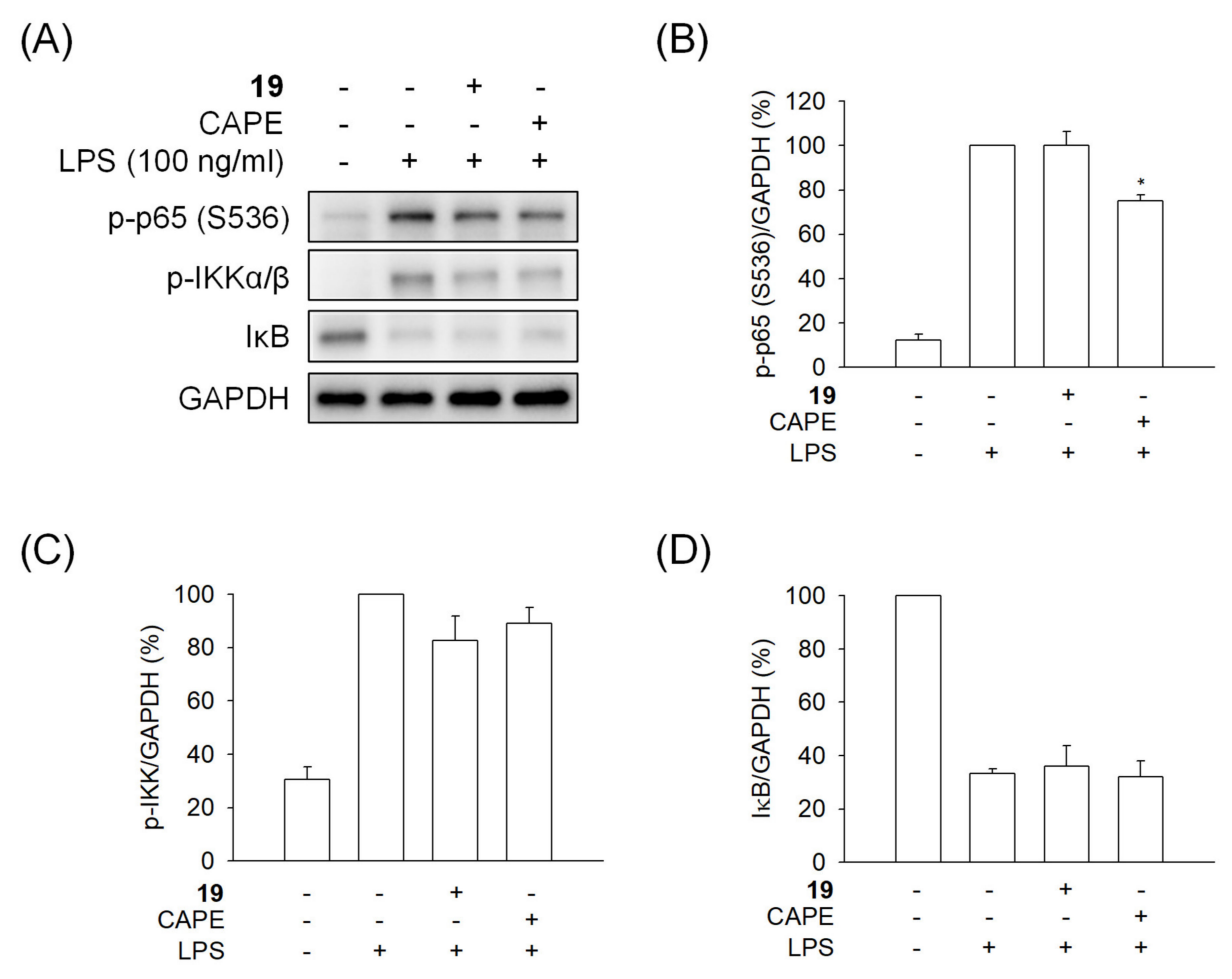
Compound **19** had no effects on IKKα/β-IκB signaling in LPS-activated dTHP−1 cells. The dTHP−1 cells were incubated with DMSO (0.1%), **19** (7.5 µM), or CAPE (10 µM) in medium for 3 h and then stimulated with or without LPS (0.1 µg/mL) for another 30 min. (**A**) The levels of phospho-NF-κB p65 (S536), phospho-IKKα/β, IκB, and GAPDH were determined by Western blot. The normalized (**B**) phospho-NF-κB p65 (S536), (**C**) phospho-IKKα/β, and (**D**) IκB levels were quantified. All data are expressed as mean ± SEM (*n* = 3); * *p* < 0.05 compared with the DMSO + LPS group.

To analyze these crucial NF-κB signaling components, dTHP−1 cells were treated by **19**, stimulated by LPS, and the cell proteins isolated and subjected to Western blot analysis. The results are shown in Figure 5. It turned out that **19** neither blocked the activation of IKKα/β nor the subsequent degradation of IκB (Figure 5A,C,D). Furthermore, the phosphorylation of p65 at Ser536 was not affected by **19** either (Figure 5A,B). Thus, it could be concluded that, unlike the established IKK inhibitors, **19** did not block the release of the NF-κB dimer.

In contrast, the pleiotropic agent CAPE effected a slight reduction in the p65 phosphorylation (Figure 5A,B), suggesting a partial inhibition of the kinase phosphorylating p65-Ser536 in THP−1 cells.

#### 2.5.3. Compound **19** Inhibits the Phosphorylation of p65-Ser468

So far, no evidence had emerged as to why the NF-κB was released from the IκB complex but still retained in the cytosol. Hence, we analyzed another regulatory site in p65, Ser468, because there was evidence from the literature that this site was phosphorylated only after the release of p65/RelA [45]. In Jurkat T cells, the IKK-related kinase IKKε was identified as the Ser468 kinase, and the expression of a kinase-dead IKKε mutant entailed a strong reduction in the NF-κB translocation to the nucleus [45]. Indeed, the treatment of the dTHP−1 cells with 7.5 µM of **19** led to a significantly diminished phosphorylation of p65-Ser468 (Figure 4C,D), suggesting that this inhibitory effect could be related to the retention of the NF-κB dimer in the cytoplasm (see Figure 1 for illustration).

#### 2.5.4. Compound **19** Has No Effect on the Kinases Involved in NF-κB Activation

Since we found that **19** led to a reduction in the Ser468 phosphorylation on p65/RelA, it was straightforward to explore whether **19** was directly inhibiting a kinase involved in NF-κB activation. This was a theoretical possibility, although our original dual EGFR/NF-κB inhibitor had not significantly affected any kinase other than EGFR in a panel of 106 kinases representing all the branches of the kinome [35]. While the EGFR kinase inhibitory activity was largely abolished here, it could not be excluded that our modifications may have generated affinity to another kinase. The most important kinases to be checked were those reported to phosphorylate p65-Ser468 in different cell types, comprising GSK3β, IKKβ, and IKKɛ [45,46,47]. In addition, all the kinases described in the literature to control the canonical and non-canonical pathway of NF-κB activation were also included in the screening panel. The screening revealed that **19** did not appreciably inhibit any of these kinases (Table S1). Hence, it was rather unlikely that a kinase was targeted by **19** directly, unless it was a kinase whose role in the NF-κB activation pathway is as yet unknown. Instead, the inhibitory effect of **19** on the Ser468 phosphorylation might be indirect, e.g., through interference with proteins complexes regulating the Ser468 kinase activity.

## 3. Materials and Methods

### 3.1. Chemistry

Solvents and reagents were obtained from commercial suppliers and used as received. Melting points were determined on a Stuart SMP3 melting point apparatus. All final compounds had a percentage purity of at least 95%, as measured by HPLC. A SpectraSYSTEM (ThermoFisher Scientific, Waltham, MA, USA) or an Ultimate 3000 (ThermoFisher Scientific) LC system was used, either consisting of a pump, an autosampler, and a UV detector. Mass spectrometry was performed on an MSQ plus electrospray mass spectrometer (ThermoFisher, Dreieich, Germany). An RP C18 column was used as the stationary phase. Three different methods were used in which the solvent system consisted of water containing 0.1% TFA or FA (A) and 0.1% TFA or FA in acetonitrile (B). HPLC Method 1: flow rate 1 mL/min; the percentage of B started at an initial of 5%, was increased up to 100% during 15 min, kept at 100% for 5 min, and flushed to 5% in 4 min and maintained for 1 min. Method 2: flow rate 0.9 mL/min; the percentage of B started at an initial of 5%, was increased up to 100% during 10 min, kept at 100% for 1 min, and flushed to 5% in 1 min. Method 3: flow rate 0.7 mL/min; the percentage of B started at an initial of 5% for 2 min, was increased to 98% during 6 min, kept at 98% for 2 min, and flushed to 5% in 2 min. Chemical shifts were recorded as δ values in ppm units and referenced against the residual solvent peak (DMSO-*d*_6_, δ = 2.50). Splitting patterns describe apparent multiplicities and are designated as s (singlet), brs (broad singlet), d (doublet), dd (doublet of doublet), t (triplet), q (quartet), m (multiplet). Coupling constants (J) are given in hertz (Hz).

#### General Synthetic Procedures and Experimental Details

Procedure A, procedure for synthesis of compounds **b2**–**c2**.

2-Amino-5-nitrobenzonitrile (5 g, 30.6 mmol) was held at reflux in triethyl orthoformate (50 mL) for 16 h in the presence of acetic anhydride (10 drops). The reaction was then concentrated under vacuum, and the remaining residue was poured onto ice water, at which point a precipitate formed. The precipitate was filtered under vacuum and left to dry to provide compound **a**. Compound **a** (1.1 g, 5 mmol) was held at reflux for 1 h with the corresponding aniline derivative in 8 mL glacial acetic acid. A precipitate was formed during the reaction, which was filtered while hot, and the precipitate was then washed with Et_2_O to provide the corresponding nitroquinazoline derivatives (**b1**–**c1**). Consequently, the desired nitroquinazoline derivative **(b1**–**c1)** (5 mmol) was mixed with stannous chloride (5.625 gm, 25 mmol) in MeOH (20 mL), and then the mixture was stirred at reflux for 30 min under a nitrogen atmosphere. Excess MeOH was removed under reduced pressure; the remaining residue was dissolved in EtOAc (200 mL) and made alkaline with an aqueous solution of NaHCO_3_. The resulting mixture was filtrated under vacuum followed by separation of the organic phase from the aqueous phase. The aqueous phase was extracted with EtOAc (2 × 20 mL), the organic fractions were combined, dried over anhydrous MgSO_4_, and concentrated under reduced pressure to obtain the corresponding aminoquinazoline derivative **(b2**–**c2)**.

Procedure B, procedure for synthesis of compounds **1**–**11** and **17**–**20**.

The selected aminoquizaoline derivative **b2** (2 mmol) was added to water (20 mL), into which concentrated HCl (1 mL) was then added and stirred at 0 °C. Thiophosgene (0.253 gm, 2.2 mmol) was then added dropwise in a well-ventilated hood to the stirred solution; stirring continued for 3 h, after which the formed precipitate was filtered and washed with Et_2_O to provide compound **b3**. Afterwards, a mixture of the isothiocyanate derivative (1 mmol) and the corresponding amine derivative (1 mmol) was stirred at room temperature for 5 h in DMF (10 mL). The solution was then poured onto ice water, at which point a precipitate formed that was then filtered. The solid was then purified by column chromatography to provide the final compounds.

Procedure C, procedure for synthesis of compounds **12**–**16**.

The desired aminoquinazoline derivative **b2 or c2** (1 mmol) and the corresponding isocyanate or isothiocyanate derivative (1 mmol) were stirred at room temperature for 5 h in DMF (10 mL). The solution was then poured onto ice water, at which point a precipitate formed that was then filtered. The solid was then purified by column chromatography to provide the corresponding thiourea derivatives.

N^4^-(3-Chlorophenyl)quinazoline-4,6-diamine (**b2**)

The compound was synthesized according to procedure **A** using 3-chloroaniline. Yield 81%; ^1^H NMR (300 MHz, DMSO-*d*_6_) δ 9.45 (s, 1H), 8.39 (s, 1H), 8.12 (t, *J* = 1.9 Hz, 1H), 7.84 (dd, *J* = 8.2, 1.2 Hz, 1H), 7.56 (d, *J* = 8.9 Hz, 1H), 7.43– 7.33 (m, 2H), 7.27 (dd, *J* = 8.9, 2.3 Hz, 1H), 7.09 (dd, *J* = 7.6, 1.7 Hz, 1H), 5.62 (s, 2H); ^13^C NMR (75 MHz, DMSO-*d*_6_) δ 156.17, 150.02, 147.87, 143.21, 142.09, 133.16, 130.41, 129.23, 124.33, 122.66, 121.06, 120.08, 117.20, 101.32; MS (+ESI): *m/z* = 270.89 (M + H)^+^

1-(4-(tert-Butyl)benzyl)-3-(4-((3-chlorophenyl)amino)quinazolin-6-yl)thiourea (**1**)

The compound was synthesized according to procedure **B** using **b3** and (4-(*tert*-butyl)phenyl)methanamine. Yield 48%; m.p. 203–205 °C; ^1^H NMR (500 MHz, DMSO-*d*_6_) δ 9.87 (d, *J* = 6.2 Hz, 2H), 8.64 (s, 1H), 8.47 (s, 1H), 8.36 (s, 1H), 8.12 (t, *J* = 1.9 Hz, 1H), 7.87 (dd, *J* = 8.2, 1.0 Hz, 1H), 7.82 (dd, *J* = 8.9, 2.1 Hz, 1H), 7.78 (d, *J* = 8.8 Hz, 1H), 7.43 (t, *J* = 8.1 Hz, 1H), 7.30 (q, *J* = 8.5 Hz, 4H), 7.17 (ddd, *J* = 8.1, 2.1, 0.8 Hz, 1H), 4.72 (d, *J* = 4.9 Hz, 2H), 1.23 (s, 9H); ^13^C NMR (126 MHz, DMSO-*d*_6_) δ 181.34, 157.15, 153.58, 149.31, 147.29, 140.92, 137.01, 135.83, 132.79, 131.70, 130.13, 128.16, 127.31, 124.95, 123.00, 121.02, 119.99, 117.67, 115.28, 47.40, 34.13, 31.12. MS (+ESI): *m/z* = 476.15 (M + H)^+^.

1-(4-((3-Chlorophenyl)amino)quinazolin-6-yl)-3-(4-methoxybenzyl)thiourea (**2**)

The compound was synthesized according to procedure **B** using **b3** and (4-methoxyphenyl)methanamine. Yield 52%; m.p. 165–167 °C; ^1^H NMR (500 MHz, DMSO-*d*_6_) δ 9.83 (d, *J* = 8.4 Hz, 2H), 8.64 (s, 1H), 8.45 (s, 1H), 8.32 (s, 1H), 8.13 (t, *J* = 1.9 Hz, 1H), 7.85 (ddd, *J* = 11.0, 8.6, 1.6 Hz, 2H), 7.78 (d, *J* = 8.8 Hz, 1H), 7.42 (t, *J* = 8.1 Hz, 1H), 7.30 (d, *J* = 8.6 Hz, 2H), 7.17 (dd, *J* = 7.9, 1.4 Hz, 1H), 6.88 (d, *J* = 8.5 Hz, 2H), 4.70 (d, *J* = 4.3 Hz, 2H), 3.71 (s, 3H). ^13^C NMR (126 MHz, DMSO-*d*_6_) δ 181.28, 158.32, 157.15, 153.63, 147.41, 140.94, 137.05, 132.78, 131.78, 130.83, 130.14, 128.89, 128.18, 123.00, 121.07, 120.04, 117.67, 115.27, 113.63, 55.05, 47.05. MS (+ESI): m/z = 450.05 (M + H) ^+^.

1-(4-((3-Chlorophenyl)amino)quinazolin-6-yl)-3-(4-(trifluoromethyl)benzyl)thiourea (**3**)

The compound was synthesized according to procedure **B** using **b3** and (4-(trifluoromethyl)phenyl)methanamine. Yield 25%; m.p. 165–167 °C; ^1^H NMR (500 MHz, DMSO-*d*_6_) δ 10.06 (s, 1H), 9.97 (s, 1H), 8.67 (s, 1H), 8.50 (s, 2H), 8.11 (s, 1H), 7.85 (dd, *J* = 8.8, 1.9 Hz, 2H), 7.80 (d, *J* = 8.8 Hz, 1H), 7.68 (d, *J* = 7.8 Hz, 2H), 7.56 (d, *J* = 8.0 Hz, 2H), 7.43 (t, *J* = 8.1 Hz, 1H), 7.19 (ddd, *J* = 7.9, 1.9, 0.7 Hz, 1H), 4.87 (d, *J* = 5.0 Hz, 2H). MS (+ESI): *m/z* = 488.08 (M + H) ^+^.

1-(4-Chlorobenzyl)-3-(4-((3-chlorophenyl)amino)quinazolin-6-yl)thiourea (**4**)

The compound was synthesized according to procedure **B** using **b3** and (4-chlorophenyl)methanamine. Yield 58%; m.p. 166–168 °C; ^1^H NMR (500 MHz, DMSO-*d*_6_) δ 9.95 (s, 1H), 9.85 (s, 1H), 8.65 (s, 1H), 8.46 (s, 1H), 8.42 (s, 1H), 8.13 (t, *J* = 1.8 Hz, 1H), 7.86 (dd, *J* = 8.2, 1.1 Hz, 1H), 7.82 (dd, *J* = 8.9, 2.0 Hz, 1H), 7.79 (d, *J* = 8.8 Hz, 1H), 7.43 (t, *J* = 8.1 Hz, 1H), 7.38 (s, 4H), 7.17 (dd, *J* = 7.9, 1.4 Hz, 1H), 4.77 (d, *J* = 5.2 Hz, 2H). ^13^C NMR (126 MHz, DMSO-*d*_6_) δ 181.54, 157.17, 153.70, 147.45, 140.89, 138.21, 136.83, 132.78, 131.83, 131.33, 130.15, 129.27, 128.28, 128.13, 123.05, 121.09, 120.06, 117.95, 115.29, 46.79. MS (+ESI): *m/z* = 454.03 (M + H) ^+^.

1-(4-((3-Chlorophenyl)amino)quinazolin-6-yl)-3-(4-fluorobenzyl)thiourea (**5**)

The compound was synthesized according to procedure B using **b3** and (4-fluorophenyl)methanamine. Yield 44%; m.p. 164–166 °C; ^1^H NMR (500 MHz, DMSO) δ 9.91 (s, 1H), 9.84 (s, 1H), 8.64 (s, 1H), 8.46 (s, 1H), 8.40 (s, 1H), 8.13 (t, *J* = 1.9 Hz, 1H), 7.86 (dd, *J* = 8.2, 1.1 Hz, 1H), 7.83 (dd, *J* = 8.9, 1.9 Hz, 1H), 7.79 (d, *J* = 8.8 Hz, 1H), 7.43–7.38 (m, 3H), 7.19–7.12 (m, 3H), 4.76 (d, *J* = 4.6 Hz, 2H); ^13^C NMR (126 MHz, DMSO-*d*_6_) δ 181.48, 161.21 (d, *J* = 242.5 Hz), 157.15, 153.71, 147.54, 140.92, 136.87, 135.28, 132.77, 131.83, 130.14, 129.44 (d, *J* = 8.1 Hz), 128.30, 123.01, 121.07, 120.04, 117.91, 115.29, 114.91 (d, *J* = 21.2 Hz), 46.74; MS (+ESI): *m/z* = 438.01 (M + H) ^+^.

1-(4-((3-chlorophenyl)amino)quinazolin-6-yl)-3-(2,4-difluorobenzyl)thiourea (**6**)

The compound was synthesized according to procedure **B** using **b3** and (2,4-difluorophenyl)methanamine. Yield 33%; m.p. 178–180 °C; ^1^H NMR (500 MHz, DMSO-*d*_6_) δ 9.96 (s, 1H), 9.84 (s, 1H), 8.64 (s, 1H), 8.47 (s, 1H), 8.38 (s, 1H), 8.13 (s, 1H), 7.85 (dd, *J* = 11.2, 4.4 Hz, 2H), 7.79 (d, *J* = 8.8 Hz, 1H), 7.45 (dt, *J* = 16.3, 8.4 Hz, 2H), 7.24–7.19 (m, 1H), 7.17 (dd, *J* = 7.9, 1.4 Hz, 1H), 7.07 (t, *J* = 7.7 Hz, 1H), 4.77 (d, *J* = 5.0 Hz, 2H); ^13^C NMR (126 MHz, DMSO-*d*_6_) δ 181.74, 161.36 (dd, *J* = 245.2, 12.1 Hz), 159.92 (dd, *J* = 247.1, 12.3 Hz), 157.16, 153.74, 147.55, 140.91, 136.88, 132.77, 131.84, 130.69, 130.15, 128.28, 123.03, 122.12 (dd, *J* = 14.7, 2.7 Hz), 121.09, 120.06, 117.92, 115.27, 111.18 (dd, *J* = 21.1, 3.2 Hz), 103.62 (t, *J* = 25.8 Hz), 40.87; MS (+ESI): *m/z* = 456.05 (M + H)^+^.

1-(4-((3-Chlorophenyl)amino)quinazolin-6-yl)-3-(2,4-dimethoxybenzyl)thiourea (**7**)

The compound was synthesized according to procedure **B** using **b3** and (2,4-dimethoxyphenyl)methanamine. Yield 43%; m.p. 204–206 °C. ^1^H NMR (500 MHz, DMSO) δ 9.82 (s, 2H), 8.63 (s, 1H), 8.45 (s, 1H), 8.12 (s, 1H), 8.07 (s, 1H), 7.90 (d, *J* = 8.8 Hz, 1H), 7.85 (d, *J* = 8.1 Hz, 1H), 7.78 (d, *J* = 8.8 Hz, 1H), 7.42 (t, *J* = 8.1 Hz, 1H), 7.18 (dd, *J* = 16.0, 8.1 Hz, 2H), 6.57 (s, 1H), 6.49 (d, *J* = 7.6 Hz, 1H), 4.63 (s, 2H), 3.80 (s, 3H), 3.74 (s, 3H). ^13^C NMR (126 MHz, DMSO) δ 181.36, 159.91, 157.83, 157.11, 153.61, 147.42, 140.96, 137.28, 132.76, 131.73, 130.12, 129.30, 128.07, 122.98, 121.09, 120.06, 118.27, 117.32, 115.22, 104.26, 98.29, 55.46, 55.22, 42.73. MS (+ESI): *m/z* = 480.11 (M + H)^+^.

1-(4-((3-Chlorophenyl)amino)quinazolin-6-yl)-3-(3-methoxybenzyl)thiourea (**8**)

The compound was synthesized according to procedure **B** using **b3** and (3-methoxyphenyl)methanamine. Yield 54%; m.p. 183–185 °C; ^1^H NMR (500 MHz, DMSO) δ 9.87 (d, *J* = 14.5 Hz, 2H), 8.64 (s, 1 H), 8.46 (s, 1H), 8.37 (s, 1H), 8.12 (s, 1H), 7.88–7.81 (m, 2H), 7.79 (d, *J* = 8.8 Hz, 1H), 7.42 (t, *J* = 8.1 Hz, 1H), 7.24 (t, *J* = 7.8 Hz, 1H), 7.17 (dd, *J* = 7.9, 1.8 Hz, 1H), 6.93 (d, *J* = 7.4 Hz, 2H), 6.84–6.78 (m, 1H), 4.75 (d, *J* = 4.4 Hz, 2H), 3.73 (s, 3H); ^13^C NMR (126 MHz, DMSO) δ 181.55, 159.27, 157.17, 153.66, 147.41, 140.90, 140.59, 137.01, 132.76, 131.87, 130.13, 129.30, 128.17, 123.04, 121.14, 120.11, 119.61, 117.85, 115.27, 113.23, 112.10, 54.99, 47.49. MS (+ESI): *m/z* = 450.04 (M + H)^+^.

1-(4-((3-Chlorophenyl)amino)quinazolin-6-yl)-3-(thiophen-2-yl-methyl)thiourea (**9**)

The compound was synthesized according to procedure **B** using **b3** and thiophen-2-ylmethanamine. Yield 60%; mp 178.8–180.7 °C; ^1^H NMR (500 MHz, DMSO-*d*_6_) δ 9.89 (s, 1H), 9.83 (s, 1H), 8.64 (s, 1H), 8.43 (d, *J* = 2.0 Hz, 2H), 8.13 (t, *J* = 2.0 Hz, 1H), 7.86 (ddd, *J* = 8.3, 2.1, 0.9 Hz, 1H), 7.82 (dd, *J* = 8.9, 2.1 Hz, 1H), 7.78 (d, *J* = 8.8 Hz, 1H), 7.42 (t, *J* = 8.1 Hz, 1H), 7.39 (dd, *J* = 5.1, 1.2 Hz, 1H), 7.17 (ddd, *J* = 8.0, 2.1, 0.9 Hz, 1H), 7.07 (dd, *J* = 3.4, 1.1 Hz, 1H), 6.96 (dd, *J* = 5.1, 3.4 Hz, 1H), 4.95 (d, *J* = 5.5 Hz, 2H); ^13^C NMR (126 MHz, DMSO-*d*_6_) δ 181.08, 157.13, 153.72, 147.59, 141.62, 140.91, 136.82, 132.76, 131.82, 130.11, 128.26, 126.47, 126.05, 125.19, 122.98, 121.07, 120.04, 117.88, 115.27, 42.56; MS (ESI) *m/z* = 425.65 (M + H)^+^.

1-(4-((3-Chlorophenyl)amino)quinazolin-6-yl)-3-(furan-2-ylmethyl)thiourea (**10**)

The compound was synthesized according to procedure **B** using **b3** and furan-2-ylmethanamine. Yield 14%; mp 195.6–198.2 °C; ^1^H NMR (500 MHz, DMSO-*d*_6_) δ 9.83 (d, *J* = 5.0 Hz, 2H), 8.64 (s, 1H), 8.45 (d, *J* = 2.1 Hz, 1H), 8.33 (s, 1H), 8.12 (t, *J* = 2.0 Hz, 1H), 7.88–7.82 (m, 2H), 7.78 (d, *J* = 8.8 Hz, 1H), 7.60 (d, *J* = 0.9 Hz, 1H), 7.42 (t, *J* = 8.1 Hz, 1H), 7.25–7.10 (m, 1H), 6.42 (dd, *J* = 3.1, 1.9 Hz, 1H), 6.35 (d, *J* = 3.0 Hz, 1H), 4.77 (d, *J* = 4.8 Hz, 2H); ^13^C NMR (126 MHz, DMSO-*d*_6_) δ 181.48, 157.14, 153.70, 151.70, 147.56, 142.16, 140.92, 137.03, 132.75, 131.84, 130.11, 128.13, 122.99, 121.11, 120.08, 117.86, 115.21, 110.51, 107.40, 40.92; MS (ESI) *m/z* = 409.75 (M + H)^+^.

1-(4-((3-Chlorophenyl)amino)quinazolin-6-yl)-3-(pyridin-3-ylmethyl)thiourea (**11**)

The compound was synthesized according to procedure **B** using **b3** and pyridin-3-ylmethanamine. Yield 11%; mp 205.6–207.9 °C; ^1^H NMR (500 MHz, DMSO-*d*_6_) δ 9.98 (s, 1H), 9.84 (s, 1H), 8.65 (s, 1H), 8.57 (d, *J* = 1.6 Hz, 1H), 8.52–8.37 (m, 3H), 8.13 (t, *J* = 1.9 Hz, 1H), 7.86 (dd, *J* = 8.3, 1.1 Hz, 1H), 7.83–7.74 (m, 3H), 7.42 (t, *J* = 8.1 Hz, 1H), 7.35 (dd, *J* = 7.7, 4.7 Hz, 1H), 7.17 (ddd, *J* = 8.0, 2.0, 0.8 Hz, 1H), 4.80 (d, *J* = 5.4 Hz, 2H); ^13^C NMR (126 MHz, DMSO-*d*_6_) δ 181.60, 157.15, 153.76, 148.93, 148.05, 147.62, 140.89, 136.70, 135.22, 134.65, 132.76, 131.78, 130.12, 128.39, 123.33, 123.02, 121.11, 120.07, 117.98, 115.30, 45.12; MS (ESI) *m/z* = 420.74 (M + H)^+^.

1-(3-Chlorobenzyl)-3-(4-((3-chlorophenyl)amino)quinazolin-6-yl)urea (**12**)

The compound was synthesized according to procedure **C** using **b2** and 1-chloro-3-(isocyanatomethyl)benzene. Yield 32%; mp 225.5–227 °C; ^1^H NMR (500 MHz, DMSO-*d*_6_) δ 9.80 (s, 1H), 8.96 (s, 1H), 8.54 (s, 1H), 8.43 (d, *J* = 2.2 Hz, 1H), 8.06 (t, *J* = 2.0 Hz, 1H), 7.86 (dd, *J* = 9.0, 2.3 Hz, 1H), 7.80 (ddd, *J* = 8.3, 2.1, 0.9 Hz, 1H), 7.73 (d, *J* = 9.0 Hz, 1H), 7.41–7.38 (m, 2H), 7.38–7.36 (m, 1H), 7.33–7.29 (m, 2H), 7.14 (ddd, *J* = 8.0, 2.1, 0.9 Hz, 1H), 6.96 (t, *J* = 6.0 Hz, 1H), 4.37 (d, *J* = 6.0 Hz, 2H); ^13^C NMR (126 MHz, DMSO-*d*_6_) δ 156.97, 155.19, 152.21, 145.61, 143.09, 141.19, 138.46, 133.01, 132.66, 130.22, 129.97, 128.37, 126.85, 126.67, 126.44, 125.78, 122.75, 121.27, 120.27, 115.71, 109.30, 42.29; MS (ESI) *m/z* = 437.76 (M + H)^+^.

1-(4-((3-Chlorophenyl)amino)quinazolin-6-yl)-3-(3-fluorobenzyl)urea (**13**)

The compound was synthesized according to procedure **C** using **b2** and 1-fluoro-3-(isocyanatomethyl)benzene. Yield 39%; mp 210.8–212 °C; ^1^H NMR (500 MHz, DMSO-*d*_6_) δ 9.80 (s, 1H), 8.95 (s, 1H), 8.54 (s, 1H), 8.43 (d, *J* = 2.2 Hz, 1H), 8.06 (t, *J* = 2.0 Hz, 1H), 7.87 (dd, *J* = 9.0, 2.3 Hz, 1H), 7.80 (ddd, *J* = 8.3, 2.0, 0.9 Hz, 1H), 7.73 (d, *J* = 9.0 Hz, 1H), 7.42–7.35 (m, 2H), 7.18 (d, *J* = 7.7 Hz, 1H), 7.16–7.12 (m, 2H), 7.07 (td, *J* = 8.6, 2.7 Hz, 1H), 6.95 (t, *J* = 6.0 Hz, 1H), 4.38 (d, *J* = 6.0 Hz, 2H); ^13^C NMR (126 MHz, DMSO-*d*_6_) δ 162.28 (d, *J* = 243.3 Hz), 156.97, 155.21, 152.21, 145.60, 143.50 (d, *J* = 6.9 Hz), 141.19, 138.48, 132.66, 130.27 (d, *J* = 8.3 Hz), 129.97, 128.37, 126.44, 123.04 (d, *J* = 2.5 Hz), 122.76, 121.27, 120.28, 115.71, 113.68 (d, *J* = 21.5 Hz), 113.46 (d, *J* = 20.9 Hz), 109.29, 42.34; MS (ESI) *m/z* = 421.75 (M + H)^+^.

1-(3-Chlorobenzyl)-3-(4-((3-ethynylphenyl)amino)quinazolin-6-yl)urea (**14**)

The compound was synthesized according to procedure **C** using **c2** and 1-chloro-3-(isocyanatomethyl)benzeneYield 22%; mp 222.4–228.8 °C; ^1^H NMR (500 MHz, DMSO-*d*_6_) δ 9.75 (s, 1H), 8.95 (s, 1H), 8.51 (s, 1H), 8.42 (d, *J* = 2.1 Hz, 1H), 8.02 (s, 1H), 7.94–7.82 (m, 2H), 7.72 (d, *J* = 9.0 Hz, 1H), 7.38 (td, *J* = 7.8, 2.6 Hz, 3H), 7.31 (dd, *J* = 8.3, 4.6 Hz, 2H), 7.20 (d, *J* = 7.6 Hz, 1H), 6.96 (s, 1H), 4.37 (d, *J* = 6.0 Hz, 2H), 4.18 (s, 1H).^13^C NMR (126 MHz, DMSO-*d*_6_) δ 157.08, 155.21, 152.31, 145.58, 143.10, 139.85, 138.39, 133.01, 130.23, 128.82, 128.33, 126.86, 126.67, 126.43, 126.38, 125.79, 124.86, 122.72, 121.68, 115.69, 109.39, 83.59, 80.48, 42.30; MS (ESI) *m/z* = 427.58 (M + H)^+^.

N-((4-((3-Chlorophenyl)amino)quinazolin-6-yl)carbamoyl)benzamide (**15**)

The compound was synthesized according to procedure **C** using **b2** and benzoyl isocyanate. Yield 45%; mp 252.4–254 °C; ^1^H NMR (500 MHz, DMSO-*d*_6_) δ 11.27 (s, 1H), 11.24 (s, 1H), 9.88 (s, 1H), 8.62 (s, 1H), 8.50 (d, *J* = 2.2 Hz, 1H), 8.39 (dd, *J* = 9.0, 2.2 Hz, 1H), 8.11 (t, *J* = 2.0 Hz, 1H), 8.07 (dd, *J* = 8.4, 1.2 Hz, 2H), 7.88–7.82 (m, 2H), 7.70–7.65 (m, 1H), 7.57 (t, *J* = 7.7 Hz, 2H), 7.42 (t, *J* = 8.1 Hz, 1H), 7.17 (ddd, *J* = 8.0, 2.0, 0.8 Hz, 1H); ^13^C NMR (126 MHz, DMSO-*d*_6_) δ 169.09, 157.04, 153.24, 151.40, 146.65, 140.88, 135.88, 133.21, 132.75, 132.15, 130.06, 128.76, 128.63, 128.37, 126.95, 123.05, 121.32, 120.26, 115.43, 112.13; MS (ESI) *m/z* = 417.57 (M + H)^+^.

N-((4-((3-Chlorophenyl)amino)quinazolin-6-yl)carbamothioyl)benzamide (**16**)

The compound was synthesized according to procedure **C** using **b2** and benzoyl isothiocyanate. Yield 74%; mp 186.1–188.5 °C; ^1^H NMR (500 MHz, DMSO-*d*_6_) δ 12.83 (s, 1H), 11.83 (s, 1H), 9.95 (s, 1H), 8.69 (s, 1H), 8.62 (d, *J* = 2.2 Hz, 1H), 8.28 (d, *J* = 9.0 Hz, 1H), 8.12 (s, 1H), 8.07–7.97 (m, 2H), 7.85 (d, *J* = 8.9 Hz, 2H), 7.69 (t, *J* = 7.4 Hz, 1H), 7.57 (t, *J* = 7.8 Hz, 2H), 7.43 (t, *J* = 8.1 Hz, 1H), 7.19 (ddd, *J* = 8.0, 2.1, 0.9 Hz, 1H); ^13^C NMR (126 MHz, DMSO-*d*_6_) δ 180.40, 168.60, 157.34, 154.24, 147.92, 140.68, 136.27, 133.29, 132.78, 132.04, 131.72, 130.13, 128.75, 128.52, 127.74, 123.28, 121.39, 120.30, 118.78, 115.06; MS (ESI) *m/z* = 433.70 (M + H)^+^.

1-(4-((3-Chlorophenyl)amino)quinazolin-6-yl)-3-cyclopropylthiourea (**17**)

The compound was synthesized according to procedure **B** using **b3** and cyclopropanamine. Yield 42%; mp 219.1–220.2 °C; ^1^H NMR (500 MHz, DMSO-*d*_6_) δ 9.85 (s, 1H), 9.64 (s, 1H), 8.64 (s, 1H), 8.43 (s, 1H), 8.13 (t, *J* = 2.0 Hz, 1H), 7.86 (d, *J* = 8.1 Hz, 2H), 7.76 (d, *J* = 8.8 Hz, 1H), 7.42 (t, *J* = 8.1 Hz, 1H), 7.17 (ddd, *J* = 8.0, 2.1, 0.8 Hz, 1H), 2.89 (s, 1H), 0.71 (d, *J* = 68.4 Hz, 4H); ^13^C NMR (126 MHz, DMSO-*d*_6_) δ 182.16, 157.17, 153.68, 147.61, 140.93, 137.50, 132.74, 131.69, 130.09, 127.71, 123.00, 121.20, 120.15, 116.17, 114.99, 30.69, 6.94; MS (ESI) *m/z* = 369.84 (M + H)^+^.

1-(4-((3-Chlorophenyl)amino)quinazolin-6-yl)-3-(cyclopropylmethyl)thiourea (**18**)

The compound was synthesized according to procedure **B** using **b3** and cyclopropylmethanamine. Yield 27%; mp 226.3–228 °C; ^1^H NMR (500 MHz, DMSO-*d*_6_) δ 9.82 (s, 1H), 9.72 (s, 1H), 8.63 (s, 1H), 8.43 (d, *J* = 2.1 Hz, 1H), 8.13 (t, *J* = 2.0 Hz, 1H), 8.05 (s, 1H), 7.87–7.85 (m, 1H), 7.84 (d, *J* = 2.1 Hz, 1H), 7.77 (d, *J* = 8.9 Hz, 1H), 7.41 (t, *J* = 8.1 Hz, 1H), 7.16 (ddd, *J* = 8.0, 2.1, 0.9 Hz, 1H), 3.39 (s, 2H), 1.21–1.07 (m, 1H), 0.45 (d, *J* = 6.9 Hz, 2H), 0.26 (dd, *J* = 4.7, 1.4 Hz, 2H); ^13^C NMR (126 MHz, DMSO-*d*_6_) δ 180.90, 157.10, 153.57, 147.37, 140.95, 137.25, 132.75, 131.64, 130.10, 128.11, 122.96, 121.09, 120.06, 117.24, 115.23, 48.75, 10.40, 3.31; MS (ESI) *m/z* = 383.83 (M + H)^+^.

1-(4-((3-Chlorophenyl)amino)quinazolin-6-yl)-3-cyclopentylthiourea (**19**)

The compound was synthesized according to procedure **B** using **b3** and cyclopentanamine. Yield 32%; mp 229.1–231.6 °C; ^1^H NMR (500 MHz, DMSO-*d*_6_) δ 9.84 (s, 1H), 9.55 (s, 1H), 8.63 (s, 1H), 8.42 (s, 1H), 8.12 (s, 1H), 7.98 (s, 1H), 7.88 (d, *J* = 8.7 Hz, 1H), 7.84 (d, *J* = 8.0 Hz, 1H), 7.76 (d, *J* = 8.9 Hz, 1H), 7.41 (t, *J* = 8.1 Hz, 1H), 7.16 (d, *J* = 7.8 Hz, 1H), 4.58 (s, 1H), 1.96 (d, *J* = 5.4 Hz, 2H), 1.66 (s, 2H), 1.58–1.45 (m, 4H); ^13^C NMR (126 MHz, DMSO-*d*_6_) δ 180.60, 157.12, 153.40, 147.08, 140.94, 137.59, 132.75, 131.53, 130.10, 127.64, 122.99, 121.15, 120.11, 116.76, 115.15, 55.53, 31.93, 23.39; MS (ESI) *m/z* = 397.82 (M + H)^+^.

1-(tert-Butyl)-3-(4-((3-chlorophenyl)amino)quinazolin-6-yl)thiourea (**20**)

The compound was synthesized according to procedure **B** using **b3** and *tert*-butylamine. Yield 54%; mp 177.5–179.4 °C; ^1^H NMR (500 MHz, DMSO-*d*_6_) δ 9.80 (s, 1H), 9.47 (s, 1H), 8.62 (s, 1H), 8.39 (d, *J* = 2.2 Hz, 1H), 8.12 (t, *J* = 2.0 Hz, 1H), 7.89 (dd, *J* = 8.9, 2.2 Hz, 1H), 7.84 (ddd, *J* = 8.3, 2.1, 0.9 Hz, 1H), 7.74 (d, *J* = 8.9 Hz, 1H), 7.63 (s, 1H), 7.41 (t, *J* = 8.1 Hz, 1H), 7.16 (ddd, *J* = 8.0, 2.1, 0.9 Hz, 1H), 1.51 (s, 9H); ^13^C NMR (126 MHz, DMSO-*d*_6_) δ 180.37, 157.06, 153.47, 147.27, 140.98, 137.65, 132.73, 132.36, 130.08, 127.59, 122.93, 121.14, 120.10, 117.11, 115.09, 52.86, 28.58; MS (ESI) *m/z* = 385.81 (M + H)^+^.

### 3.2. Biological Methods

#### 3.2.1. Cell Culture

The HEK-293 (ATCC Cat# CRL-1573, RRID:CVCL_0045) and human monocytic THP−1 cells (ATCC Cat# TIB-202, RRID:CVCL_0006) were, respectively, cultured in DMEM and RPMI 1640 media supplemented with 10% fetal bovine serum, 2 mM L-glutamine, and 1× Antibiotic-Antimycotic at 37 °C in a humidified incubator with 5% CO_2_. THP−1 cells were differentiated by 100 nM phorbol-12-myristate-13-acetate (PMA) for 4 h and then rested for 44 h as dTHP−1 cells.

#### 3.2.2. NF-κB Luciferase Reporter Assay

NF-κB Luciferase Reporter Vector was purchased from Signosis (Santa Clara, CA, USA) and transfected into HEK-293 cells using FuGENE HD (Promega, Fitchburg, MA, USA) following the manufacturer’s protocol. The transfected cells were incubated in serum-free DMEM for 16 h and then treated with DMSO or indicated compounds. After 3 h, cells were treated with or without 50 ng/mL of Recombinant Human TNFα Protein (R&D systems, Minneapolis, MN, USA) for another 3 h. The protein lysates were analyzed using Steady-Glo^®^ Luciferase Assay System (Promega, Fitchburg, MA, USA) and TECAN Infinite 200 pro (Männedorf, Switzerland).

#### 3.2.3. Cell Viability

Cells were incubated with the WST-1 reagent (Thermo Fisher Scientific, Waltham, MA, USA) at 37 °C for 2 h. The cell viability was determined by spectrophotometer at 450 nm.

#### 3.2.4. ELISA Assay

The dTHP−1 cells were treated with indicated compounds for 3 h and then activated with or without 100 ng/mL lipopolysaccharide (LPS; *E. coli* 0111:B4) for another 24 h. The levels of human IL-6 and TNFα in supernatants were measured by DuoSet^®^ ELISA Development System (R&D systems, Minneapolis, MN, USA) according to the manufacturer’s protocol.

#### 3.2.5. Immunofluorescent Staining

Cells were fixed with 4% formaldehyde and then incubated with 5% goat serum. The intracellular location of NF-κB p65 was determined using primary antibodies against NF-κB p65 (Cell Signaling Technology, Beverly, MA, USA) and FITC-conjugated secondary antibody. Nuclei were counterstained with Hoechst (1 µg/mL). Images were obtained by fluorescent microscopy (OLYMPUS IX 81; Olympus, Tokyo, Japan).

#### 3.2.6. Western Blotting

Cells were lysed in the commercial RIPA Buffer (Thermo Fisher Scientific, Waltham, MA, USA) and then centrifuged at 14,000× *g* for 20 min. The lysates were boiled in sodium dodecyl sulphate (SDS) sample buffer (62.5mMTris-HCl (pH 6.8), 4% SDS, 5% β-mercaptoethanol, 2.5 mM Na_3_VO_4_, 0.0125% bromophenol blue, 10 mM di-N-pentyl phthalate, and 8.75% glycerol), separated by SDS-polyacrylamide gel electrophoresis (PAGE), and electrophoresed onto a nitrocellulose membrane. Antibodies used in this study were phospho-NF-κB p65 (S468; catalog no. 3039), phospho-NF-κB p65 (S536; catalog no. 3033), phospho-IKKα/β (catalog no. 2697), and IκB (catalog no. 9242) from Cell Signaling Technology (Beverly, MA, USA); GAPDH from ABclonal (catalog no. A10868; Woburn, MA, USA). The labeled signals were determined using an enhanced chemiluminescence system (UVP, Upland, CA, USA). The experiments were performed at least two times in triplicate, providing basically the same results.

## 4. Conclusions

In this study, we demonstrated that our previous dual EGFR kinase/NF-κB inhibitors could be modified at the 4-aminophenyl and the thiourea function to retain only the NF-κB inhibitory activity. Several of these new inhibitors, including **19** and **20**, were active in all the assays relying on NF-κB inhibition while exhibiting low to moderate cytotoxicity in the non-immune cell lines HEK293 and A549. Our most potent compounds inhibited the production of IL-6 in the submicromolar range and, to a somewhat lower extent, also of TNFα, which are both key factors in often the same inflammatory disorders. IL-6 is a pleiotropic pro-inflammatory cytokine, which is implicated in the pathophysiology of numerous chronic inflammatory and auto-immune diseases, such as multiple sclerosis, rheumatoid arthritis, and inflammatory bowel and pulmonary diseases (see Ref. [48] for a recent review). Thus, IL-6 is a pivotal target for the development of therapeutics against complex inflammatory diseases. Monoclonal antibodies neutralizing IL-6 peptides are successfully used to treat various immunoinflammatory diseases; however, their high cost, invasive route of administration, and high rate of immunogenicity remain major limitations. Small molecules that cause a decrease in IL-6 production have been identified mostly by phenotypic screening; however, the mechanisms of action were not investigated, and many of these compounds may have pleiotropic effects [48]. TNFα, which is primarily produced by macrophages, is another pivotal inflammatory mediator in numerous chronic inflammatory and autoimmune diseases [49,50,51,52,53,54]. Similar to the targeting of IL-6, anti-TNFα mAbs are used to treat inflammatory conditions, such as rheumatoid arthritis, juvenile arthritis, inflammatory bowel diseases, and psoriasis (reviewed in Refs. [55,56]). In light of their joint pro-inflammatory activities, the reduction in both IL-6 and TNFα, along with other cytokines through the inhibition of the central transcription factor NF-κB, as demonstrated for our compounds, might be even more effective in the treatment of the chronic inflammatory diseases for which either IL-6- and TNFα-mAbs are currently used. In inflammatory bowel diseases, for instance, up to 40% of the patients do not respond to anti-TNFα treatments [13].

Moreover, while mAbs do not affect the cytokine-producing cells, several of our inhibitors, particularly **6** and **19**, selectively induced cell death in the LPS-activated macrophages, significantly stronger than the reference compound, CAPE. This could be an interesting additional effect of the novel compounds, worthy of being explored in future studies as macrophage infiltrates are especially associated with tissue damage and inflammation in metabolic syndrome [57], inflammatory brain disorders, and autoimmune diseases [57,58]. In addition, macrophages are implicated in the destabilization of atherosclerotic plaques, leading to acute coronary syndromes and sudden death. Elimination of macrophages from plaques through pharmacological intervention may, therefore, represent a promising strategy to stabilize vulnerable, rupture-prone lesions [59]. Thus, in various conditions of chronic inflammation, it could be a therapeutic advantage to selectively induce cell death in activated TNFα-producing macrophages compared to a temporary reduction in or neutralization of their secreted cytokines.

With respect to the mechanism of action, we found that **19** inhibited the p65 nuclear translocation while not affecting IκB phosphorylation and degradation. Further investigating the regulatory mechanisms that were targeted by the compounds in dTHP−1 cells, we could demonstrate that **19** caused a reduction in the Ser468 but not the Ser536 phosphorylation levels. Our results provided evidence for an independent regulation of these phosphorylation events, although activating stimuli, such as LPS and TNFα, induce both the Ser536 and Ser468 phosphorylation in different immunocompetent cell types [45,60,61,62]. Since detailed knowledge on the regulation of Ser-468 phosphorylation on p65/RelA is lacking, it was not possible in the frame of this study to conduct further research aiming at identifying the direct target protein of our compounds. However, **19** and other congeners from our study might be useful tools to analyze the role and regulation of Ser468 phosphorylation in future studies.

In conclusion, our 4-aminophenyl quinazoline thiourea compounds represent a new class of inhibitors that display a combined mode of action on cytokine release and macrophage depletion. Thus, they may have potential for the treatment of various inflammatory diseases that are exacerbated by excess activated macrophages, such as arteriosclerosis and autoimmune diseases.

## Figures and Tables

**Table 1:**
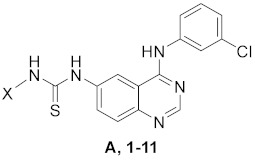
Biological activities of NF-κB inhibitors with benzylthioureido and analogous moieties.

Cpd.	X	*^a,b^* NF-κB Luciferase Activity		*^c^* Cell Viability (%)		Recombinant EGFR Kinase Activity (%inh at 150 nM)
		Inh %	IC_50_ (μM)	HEK293	A549	
**A** ^*d*^	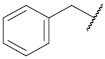	94.6 ± 1.5 **	0.79	72.4 ± 3.9 ***	81.7 ± 1.2	84.8
**1**	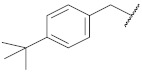	41.6 ± 3.8 ***	>10	73.9 ± 4.6 **	91.1 ± 3.4	42
**2**	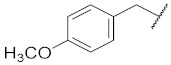	94.6 ± 0.1 ***	1.7	100.9 ± 7.4	95.4 ± 1.9	67
**3**	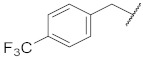	46.5 ± 4.9 ***	>10	68.9 ± 6.4 **	73.4 ± 4.3	70
**4**	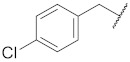	31.9 ± 9.6 **	>10	95.9 ± 3.4	98.1 ± 1.9	56
**5**	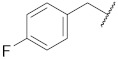	79.1 ± 0.8 ***	1.9	67.0 ± 6.1 **	65.3 ± 1.1	72
**6**	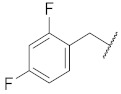	78.4 ± 2.7 **	1.6	85.5 ± 6.4 *	75.5 ± 2.9	68
**7**	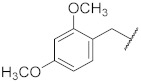	27.1 ± 3.4 ***	>10	85.9 ± 2.5 *	99.5 ± 1.4	48
**8**	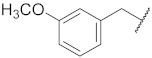	76.1 ± 2.6 ***	2.3	100.2 ± 6.9	101.1 ± 2.3	40
**9**	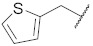	98.0 ± 0.4 ***	2.8	13.9 ± 0.7 ***	50.7 ± 2.4	69.8
**10**	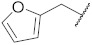	76.1 ± 2.3 ***	4.3	71.7 ± 3.1 ***	74.4 ± 11.7	79.1
**11**	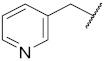	44.7 ± 5.8 **	>10	62.8 ± 4.0 ***	98.9 ± 4.1	66

*^a^* Percentage of inhibition (Inh %) at 10 μM concentration in NF-κB-luciferase-transfected HEK293 cells. Concentration necessary for 50% inhibition (IC_50_). Results are presented as mean ± SEM. (*n* = 3). * *p* < 0.05, ** *p* < 0.01, *** *p* < 0.001 compared with the DMSO + TNFα (50 ng/mL) group. *^b^* Inhibition by the reference compound CAPE at 10 µM: 72.8% (± 5.7 SEM.). *^c^* Percentage of cell viability at 10 μM concentration. Results are presented as mean ± SEM. (*n* = 3). * *p* < 0.05, ** *p* < 0.01, *** *p* < 0.001 compared with the DMSO group. *^d^* Compound published in Ref. [37].

**Table 2:**
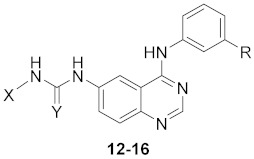
Biological activities of NF-κB inhibitors with benzyl urea and benzoyl (thio)urea moieties.

Cpd.	R	X	Y	*^a,b^* NF-κB Luciferase Activity		*^c^* Cell Viability (%)		Recombinant EGFR Kinase Activity (%inh at 150 nM)
				Inh %	IC_50_ (μM)	HEK293	A549	
**12**	Cl	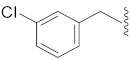	O	42.7 ± 9.3 *	>10	113.6 ± 3.2	100.7 ± 4.2	34.6
**13**	Cl	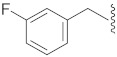	O	53.3 ± 10.0 **	8.1	95.5 ± 6.1	103.5 ± 5.0	23.3
**14**	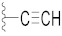	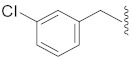	O	58.5 ± 0.9 ***	8.0	110.2 ± 2.7	98.6 ± 3.0	44.5
**15**	Cl	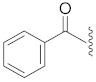	O	48.3 ± 9.3 *	>10	113.8 ± 2.6	100.1 ± 4.5	26
**16**	Cl	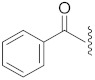	S	58.2 ± 5.2 ***	7.0	101.5 ± 4.0	102.3 ± 1.6	48.9

*^a^* Percentage of inhibition (Inh %) at 10 μM concentration in NF-κB luciferase-transfected HEK293 cells. Concentration necessary for 50% inhibition (IC_50_). Results are presented as mean ± SEM. (*n* = 3). * *p* < 0.05, ** *p* < 0.01, *** *p* < 0.001 compared with the DMSO + TNFα (50 ng/mL) group. *^b^* Inhibition by the reference compound, CAPE, at 10 µM: 72.8% (± 5.7 SEM.). *^c^* Percentage of cell viability at 10 μM concentration. Results are presented as mean ± SEM. (*n* = 3). * *p* < 0.05, ** *p* < 0.01, *** *p* < 0.001 compared with the DMSO group.

**Table 3:**
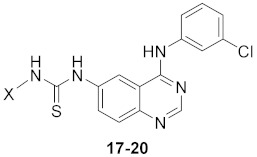
Biological activities of NF-κB inhibitors with alkylthioureido moieties.

Cpd.	X	*^a, b^* NF-κB Luciferase Activity		*^c^* Cell Viability (%)		Recombinant EGFR Kinase Activity (%inh at 150 nM)
		Inh %	IC_50_ (μM)	HEK293	A549	
**17**	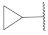	47.0 ± 5.3 ***	>10	103.0 ± 8.4	102.6 ± 0.2	36.5
**18**	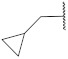	89.9 ± 1.5 ***	2.8	75.7 ± 3.8	79.1 ± 0.6	59
**19**	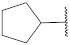	92.5 ± 0.8 ***	2.3	118.5 ± 11.4	99.9 ± 1.6	23
**20**	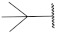	75.7 ± 3.1 **	2.0	99.7 ± 8.0	98.1 ± 3.1	1.5

*^a^* Percentage of inhibition (Inh %) at 10 μM concentration in NF-κB-luciferase-transfected HEK293 cells. Concentration necessary for 50% inhibition (IC_50_). Results are presented as mean ± SEM. (*n* = 3). ** *p* < 0.01, *** *p* < 0.001 compared with the DMSO + TNFα (50 ng/mL) group. *^b^* Inhibition by the reference compound, CAPE, at 10 µM: 72.8% (±5.7 SEM.). *^c^* Percentage of cell viability at 10 μM concentration. Results are presented as mean ± SEM. (*n* = 3). ** *p* < 0.01, *** *p* < 0.001 compared with the DMSO group.

**Table 4:** Effects of NF-κB inhibitors on TNFα and IL-6 production in LPS-activated dTHP−1 cells.

Cpd.	TNFα Production			IL-6 Production		
	Inh % (7.5 μM)	Inh % (15 μM)	IC_50_ (μM)	Inh % (7.5 μM)	Inh % (15 μM)	IC_50_ (μM)
**2**	32.5 ± 8.5 ***	60.4 ± 1.8 ***	10.2 ± 0.27	76.2 ± 13.3 **	87.5 ± 6.8 ***	1.2 ± 0.12
**6**	61.1 ± 5.8 **	76.6 ± 3.8 ***	4.4 ± 0.85	97.6 ± 0.6 ***	98.2 ± 1.3 ***	0.36 ± 0.07
**17**	19.0 ± 8.0	62.0 ± 4.2 **	12.3 ± 1.0	86.0 ± 2.3 **	95.8 ± 0.33 ***	0.72 ± 0.17
**18**	31.0 ± 4.5 **	65.9 ± 7.9 **	11.5 ± 1.7	78.9 ± 14.8 **	89.4 ± 7.0 ***	1.1 ± 0.36
**19**	82.5 ± 5.6 ***	91.5 ± 2.5 ***	4.0 ± 0.20	92.7 ± 5.5 ***	98.3 ± 0.28 ***	0.84 ± 0.12
**20**	57.4 ± 7.2 ***	89.9 ± 0.88 ***	5.1 ± 0.43	91.8 ± 3.5 ***	98.7 ± 1.2 ***	0.94 ± 0.11
**7**	37.0 ± 4.9 *	50.3 ± 5.5 *	11.9 ± 0.70	71.0 ± 3.4 **	84.0 ± 0.82 ***	1.2 ± 0.16
**CAPE**	ND	ND	11.4 ± 0.21	ND	ND	1.1 ± 0.36

Percentage of inhibition (Inh %) of 7.5 or 15 μM in LPS-activated dTHP−1 cells. Results are expressed as mean ± SEM. (*n* = 3). * *p* < 0.05, ** *p* < 0.01, *** *p* < 0.001 compared with the DMSO + LPS (100 ng/mL for 24 h) group.

**Table 5:** Effects of compounds on cell viability of LPS-activated dTHP−1 cells.

Cpd.	7.5 μM		5 μM	
	-	LPS	-	LPS
**2**	99.2 ± 1.5	57.5 ± 1.4 ***	94.2 ± 1.6 *	78.8 ± 5.3 *
**6**	62.0 ± 14.5	6.2 ± 0.12 ***	43.6 ± 9.0 **	5.0 ± 0.35 ***
**17**	94.3 ± 1.9 *	84.3 ± 2.1 **	84.5 ± 4.8 *	62.0 ± 3.4 ***
**18**	82.7 ± 10.3	34.9 ± 1.6 ***	87.6 ± 2.5 **	11.2 ± 3.3 ***
**19**	60.3 ± 0.85 ***	6.7 ± 0.14 ***	30.0 ± 1.3 ***	4.9 ± 0.38 ***
**20**	78.0 ± 0.09 ***	32.5 ± 0.89 ***	42.5 ± 10.6 **	12.5 ± 0.23 ***
**7**	94.4 ± 0.61 ***	94.7 ± 1.2 **	96.8 ± 4.2	91.9 ± 4.2
**CAPE (10 μM)**	97.8 ± 0.41 **	52.2 ± 9.7 **	ND	ND

Results are expressed as mean ± SEM. (*n* = 3). * *p* < 0.05, ** *p* < 0.01, *** *p* < 0.001 compared with the DMSO or DMSO + LPS (100 ng/mL for 24 h) group.

## Data Availability

Data is contained within the article and supplementary material.

## Supplementary Materials

The following supporting information can be downloaded at: https://www.mdpi.com/article/10.3390/ph15070778/s1, Table S1: Selectivity profiling of compound 19 against the protein kinases known to regulate the NF-κB pathway. Figure S1: ^1^H-NMR and ^13^C-NMR spectra of newly synthesized compounds.

## Abbreviations

CAPE: caffeic acid phenethyl ester; DMEM, Dulbecco’s Modified Eagle Medium; EGFR, epidermal growth factor receptor; FITC, fluorescein isothiocyanate; GAPDH, glyceraldehyde 3-phosphate dehydrogenase; GSK, glycogen synthase kinase; IκB, inhibitor of nuclear factor κB; IKK, IκB kinase; IL-6, interleukin-6; LPS, lipopolysaccharide; mAb, monoclonal antibody; NF-κB, nuclear factor ‘κ-light-chain-enhancer’ of activated B-cells; RelA, v-rel avian reticuloendotheliosis viral oncogene homolog A; RIPA, radio-immunoprecipitation assay; RPMI, Roswell Park Memorial Institute culture medium; TNFα, tumor necrosis factor α; WST1, water-soluble tetrazolium salt.
