# Supplementary material for: Development of (4-Phenylamino)quinazoline Alkylthiourea Derivatives as Novel NF-κB Inhibitors
Source: Pharmaceuticals (Basel). 2022 Jun 22;15(7):778. doi: 10.3390/ph15070778 (PMC9322122; doi:10.3390/ph15070778)
Supplement: Supplementary file 1 [file pharmaceuticals-15-00778-s001.zip › pharmaceuticals-1764568-supplementary.pdf]

Supplementary Material

Development of (4-phenylamino)quinazoline Alkylthiourea Derivatives as Novel NF-κB Inhibitors

Sarah S. Darwish [1,2,†], Po-Jen Chen [3,†], Mostafa M. Hamed [4,†], Reem A. Wagdy [1], Shun-Hua Chen [5], Ashraf H. Abadi [1], Mohammad Abdel-Halim [1], Tsong-Long Hwang [6,7,8,9,*] and Matthias Engel [10,*]

1. Department of Pharmaceutical Chemistry, Faculty of Pharmacy and Biotechnology, German University in Cairo, 11835 Cairo, Egypt; s.same@gaf.edu.eg (S.S.D.); reem.ahmed-afifi@guc.edu.eg (R.A.W.); ashraf.abadi@guc.edu.eg (A.H.A.); mohammad.abdel-halim@guc.edu.eg (M.A.-H.)
2. School of Life and Medical Sciences, University of Hertfordshire hosted by Global Academic Foundation, New Administrative Capital, 11578 Cairo, Egypt
3. Department of Medical Research, E-Da Hospital, Kaohsiung City 824005, Taiwan; litlep@hotmail.com
4. Drug design and optimization, Helmholtz Institute for Pharmaceutical Research Saarland (HIPS), Helmholtz Centre for Infection Research, Saarland University Campus, 66123 Saarbrücken, Germany; mostafa.hamed@helmholtz-hips.de
5. School of Nursing, Fooyin University, Kaohsiung 831, Taiwan; ft104@fy.edu.tw
6. Graduate Institute of Natural Products, College of Medicine, Chang Gung University, Taoyuan 333, Taiwan
7. Research Center for Chinese Herbal Medicine, Graduate Institute of Health Industry Technology, College of Human Ecology, Chang Gung University of Science and Technology, Taoyuan 333, Taiwan
8. Department of Anesthesiology, Chang Gung Memorial Hospital, Taoyuan 333, Taiwan
9. Department of Chemical Engineering, Ming Chi University of Technology, New Taipei City 243, Taiwan
10. Pharmaceutical and Medicinal Chemistry, Saarland University, Campus C2.3, 66123 Saarbrücken, Germany

† These authors contributed equally to this work.
* Correspondence: htl@mail.cgu.edu.tw (T.-L.H.); ma.engel@mx.uni-saarland.de (M.E.); Tel.: +886-3-2118506 (T.-L.H.); +49-681-302-70312 (M.E.); Fax: +886-3-2118506 (T.-L.H.); +49-681-302-70308 (M.E.)

Contents

1. Table S1
2. ^{1}H-NMR (500 MHz, DMSO) and ^{13}C-NMR (126 MHz, DMSO) spectra of newly synthesized compounds.

Table S1. Selectivity profiling of compound **19** against the protein kinases known to regulate the NF-kB pathway

**kinase**	**% Inhibition at 2 µM[a]**
	**19**
CHUK (IKK alpha)	6
IRAK1	2
MAP3K14 (NIK)	9
MAP3K7/MAP3K7IP1 (TAK1-TAB1)	3
RIPK2	29
GSK3B (GSK3 beta)	4
IKBKB (IKK beta)	3
IKBKE (IKK epsilon)	3
IRAK4	5
MAPK14 (p38 alpha)	4
MAPK3 (ERK1)	2
PRKCI (PKC iota)	n.i.
PRKCQ (PKC theta)	n.i.
PRKCZ (PKC zeta)	3
RPS6KA1 (RSK1)	1
TBK1	8

[a]Screenings were performed as a service at Thermo Fisher Scientific at an ATP concentration of 100 µM. n.i., no inhibition. Data represent mean values of duplicates that differed by less than 7%.

Figure S1 ^{1}H-NMR (500 MHz, DMSO) and ^{13}C-NMR (126 MHz, DMSO) spectra

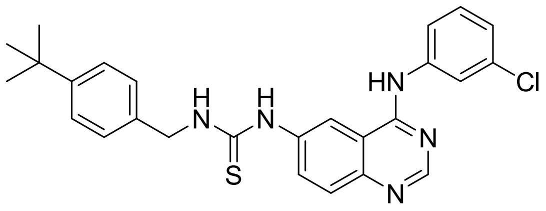

1

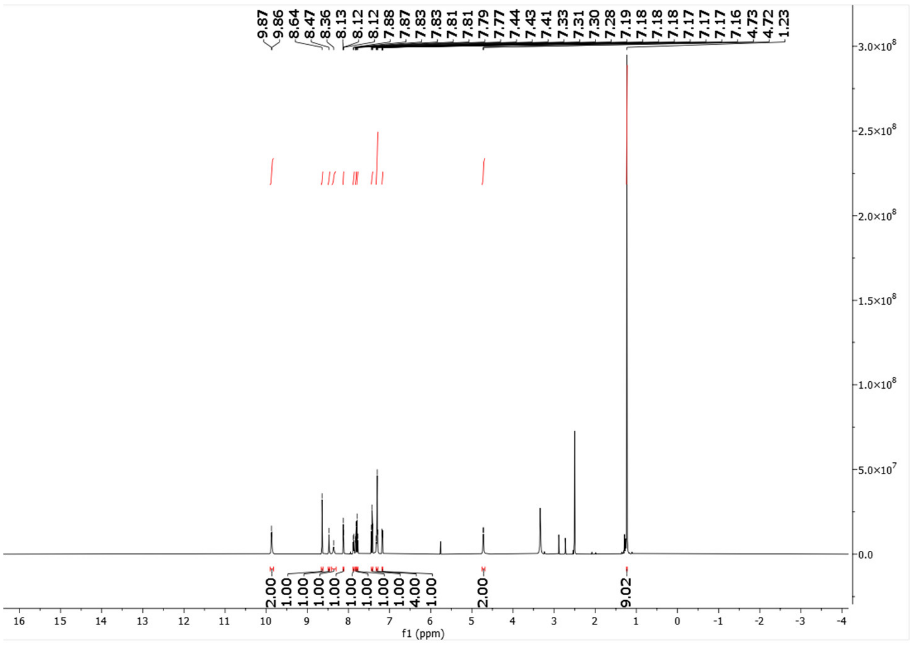

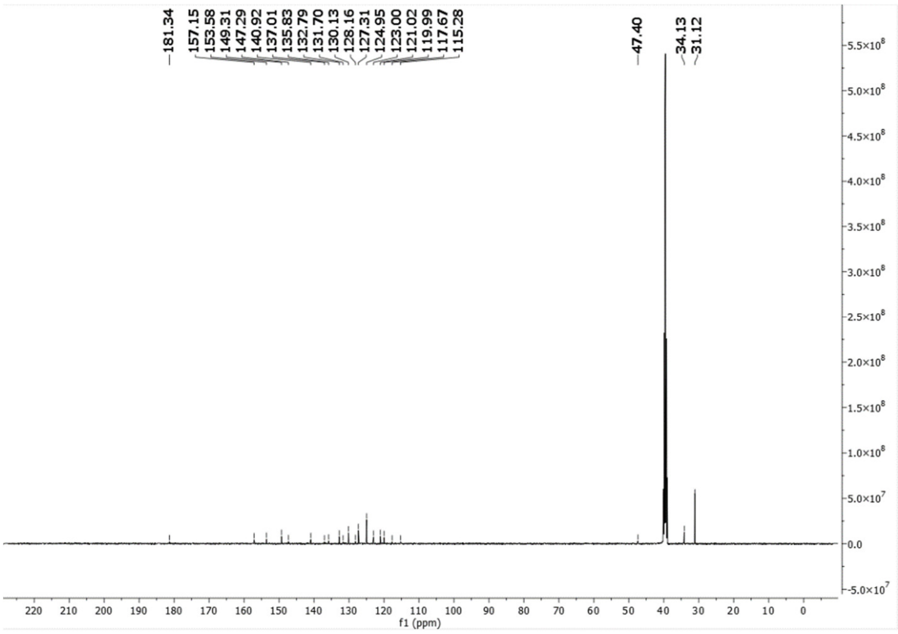

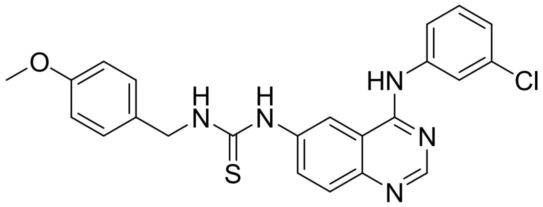

2

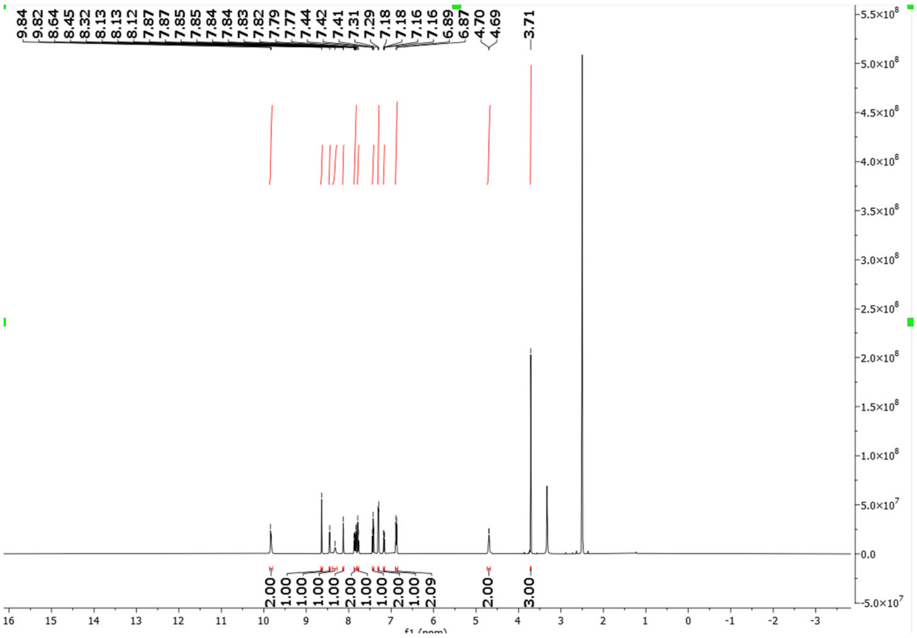

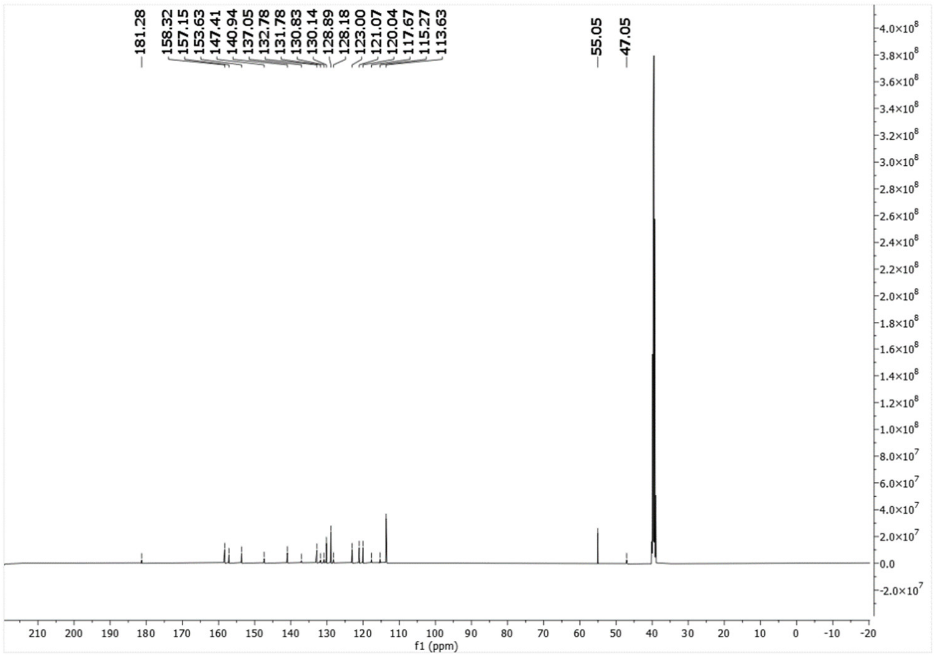

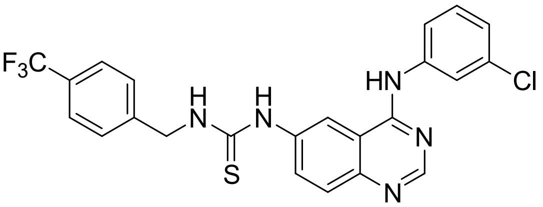

3

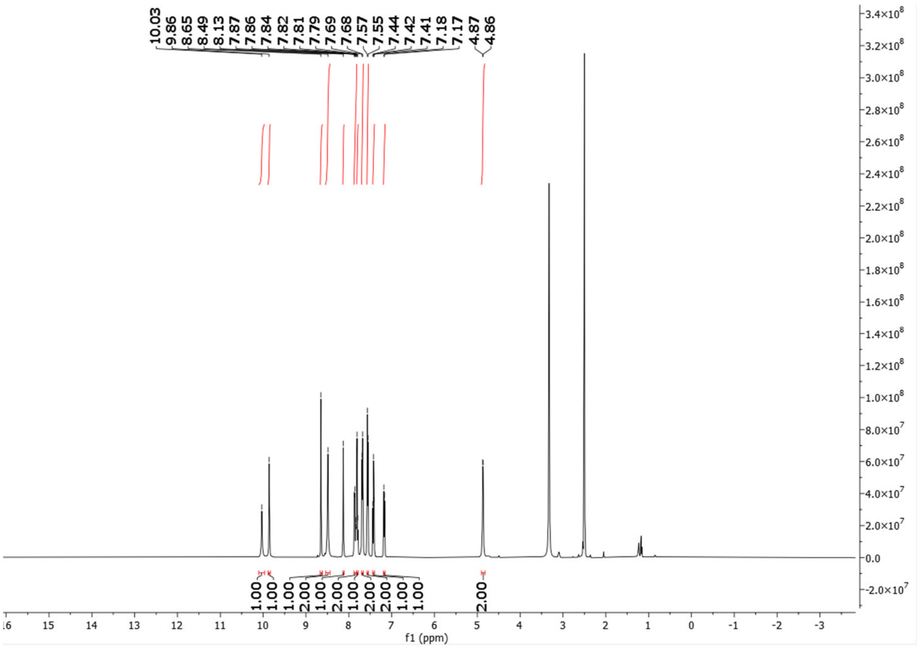

10.03
9.86
8.65
8.49
8.13
7.87
7.86
7.84
7.82
7.81
7.79
7.69
7.68
7.57
7.55
7.44
7.42
7.41
7.18
7.17
4.87
4.86
f1 (ppm)

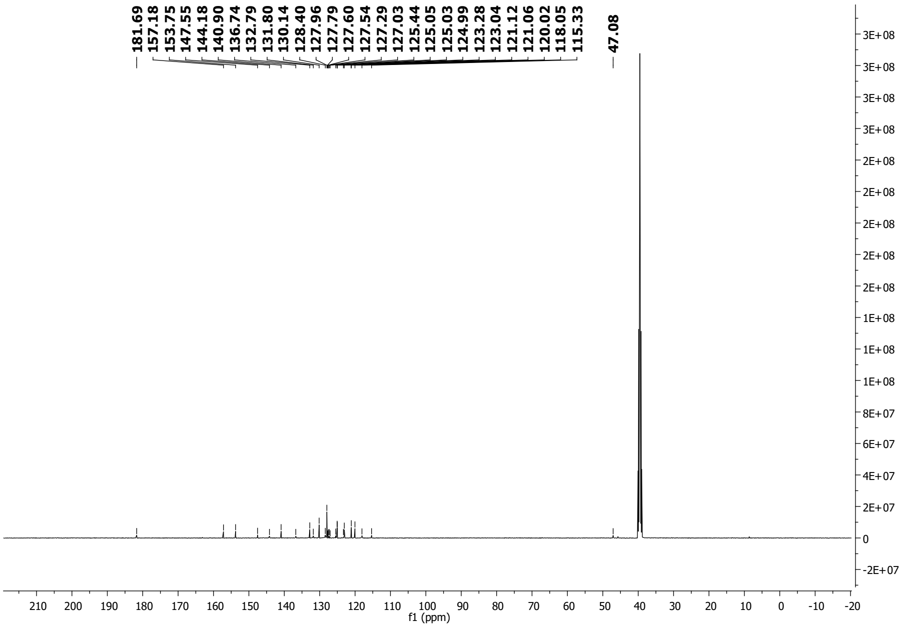

181.69
157.18
153.75
147.55
144.18
140.90
136.74
132.79
131.80
130.14
128.40
127.96
127.79
127.60
127.54
127.29
127.03
125.44
125.05
125.03
124.99
123.28
123.04
121.12
121.06
120.02
118.05
115.33
47.08
f1 (ppm)

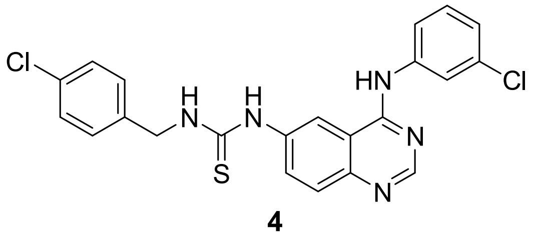
Cl
H
N
H
N
HN
Cl
N
S
N

4

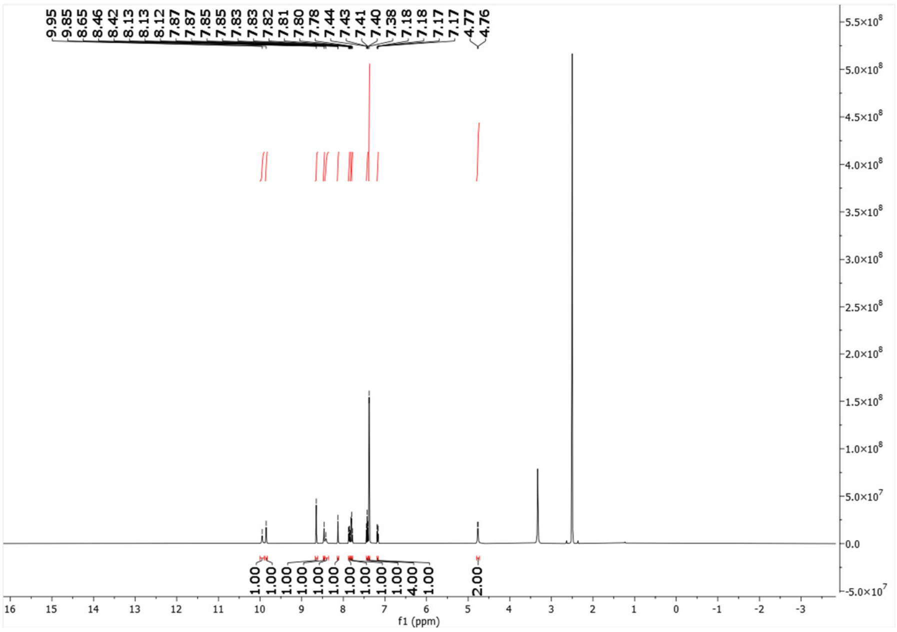
9.95
9.85
8.65
8.46
8.42
8.13
8.13
8.12
7.87
7.87
7.85
7.85
7.83
7.83
7.82
7.81
7.80
7.78
7.44
7.43
7.41
7.40
7.38
7.18
7.18
7.17
7.17
4.77
4.76
1.00
1.00
1.00
1.00
1.00
1.00
1.00
1.00
1.00
4.00
1.00
2.00
f1 (ppm)

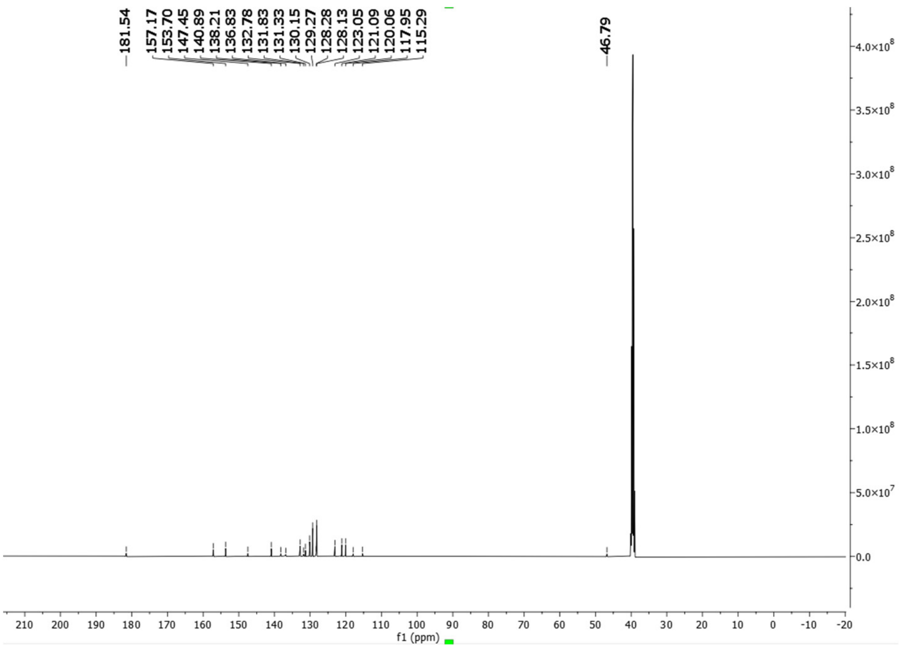
181.54
157.17
153.70
147.45
140.89
138.21
136.83
132.78
131.83
131.33
130.15
129.27
128.28
128.13
123.05
121.09
120.06
117.95
115.29
46.79
f1 (ppm)

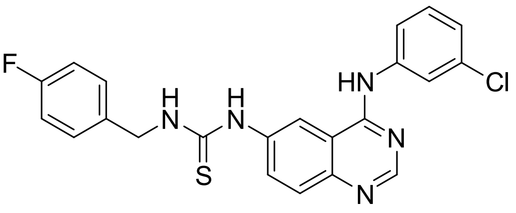

5

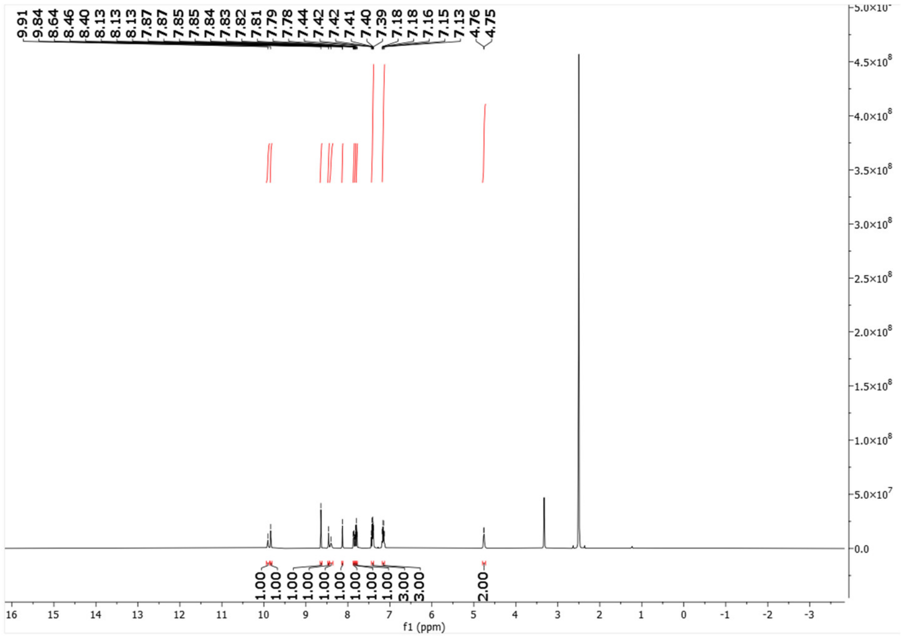
9.91 9.84 8.64 8.46 8.40 8.13 8.13 8.13 7.87 7.87 7.85 7.85 7.84 7.83 7.82 7.81 7.79 7.78 7.44 7.42 7.42 7.41 7.40 7.39 7.18 7.16 7.15 7.13 4.76 4.75
1.00 1.00 1.00 1.00 1.00 1.00 1.00 1.00 3.00 3.00 2.00
f1 (ppm)

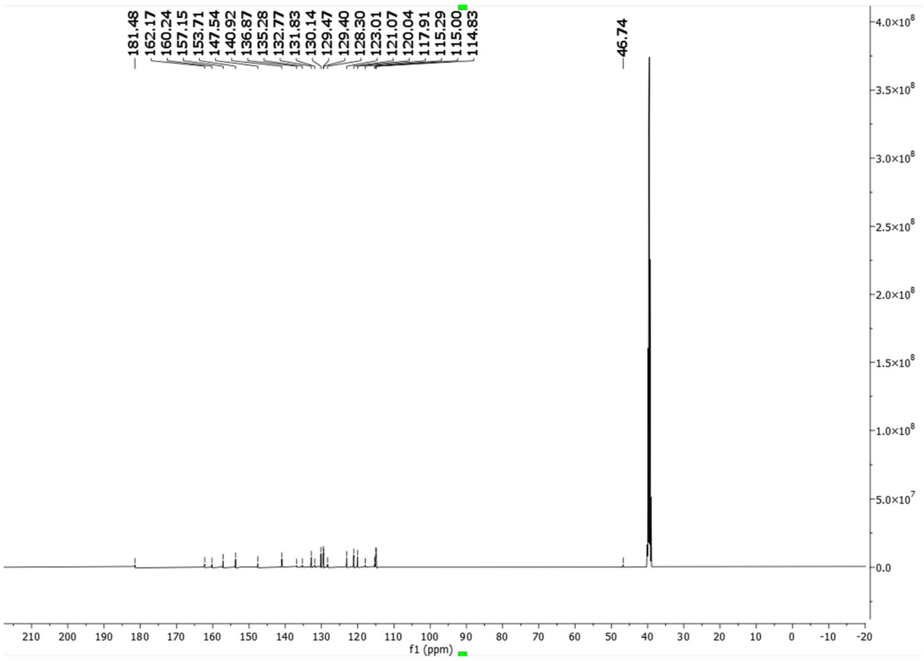
181.48 162.17 160.24 157.15 153.71 147.54 140.92 136.87 135.28 132.77 131.83 130.14 129.47 129.40 128.30 123.01 121.07 120.04 117.91 115.29 115.00 114.83
46.74
f1 (ppm)

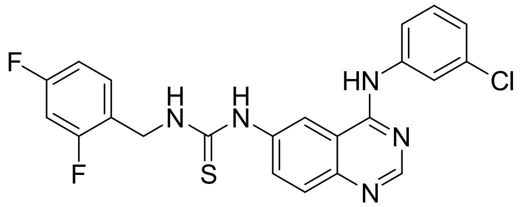
F
F
H
N
H
N
S
HN
Cl
N
N

6

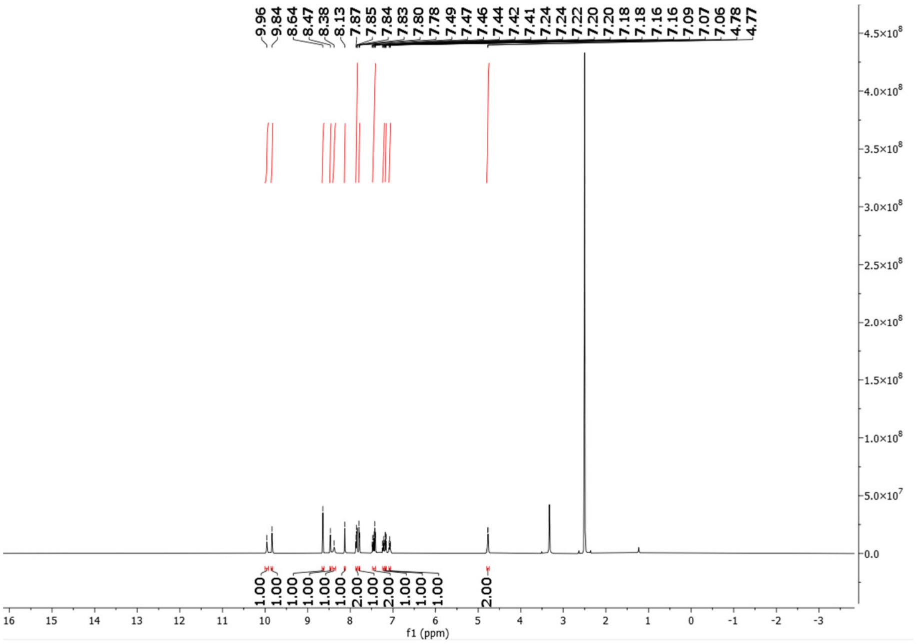
9.96
9.84
8.64
8.47
8.38
8.13
7.87
7.85
7.84
7.83
7.80
7.78
7.49
7.47
7.46
7.44
7.42
7.41
7.24
7.24
7.22
7.20
7.20
7.18
7.18
7.16
7.09
7.07
7.06
4.78
4.77
1.00
1.00
1.00
1.00
1.00
2.00
1.00
2.00
1.00
1.00
1.00
2.00
f1 (ppm)

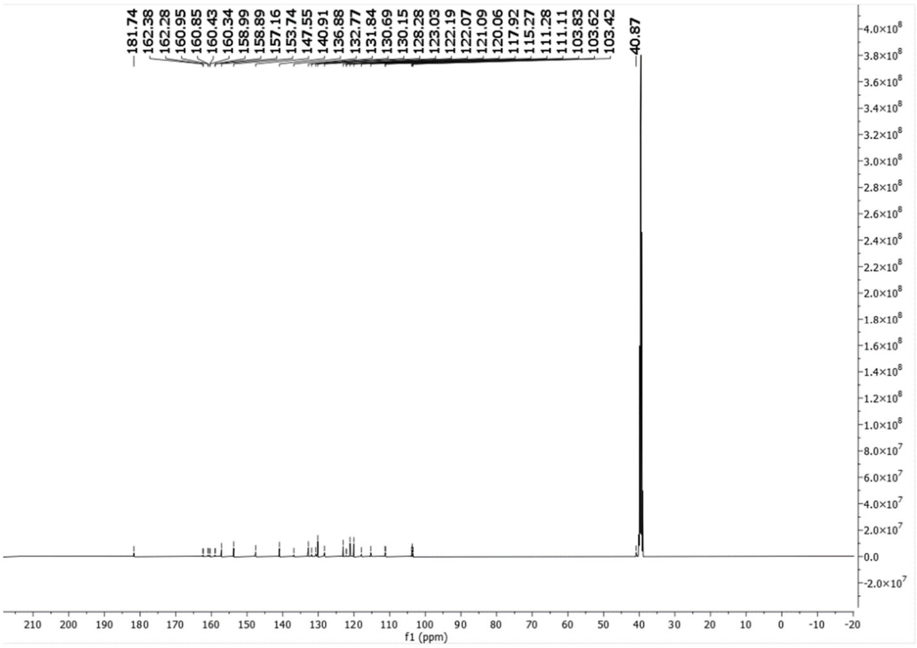
181.74
162.38
162.28
160.95
160.85
160.43
160.34
158.99
158.89
157.16
153.74
147.55
140.91
136.88
132.77
131.84
130.69
130.15
128.28
123.03
122.19
122.07
121.09
120.06
117.92
115.27
111.28
111.11
103.83
103.62
103.42
40.87
f1 (ppm)

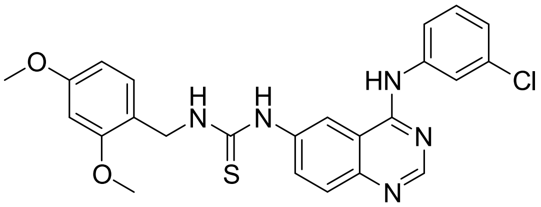

7

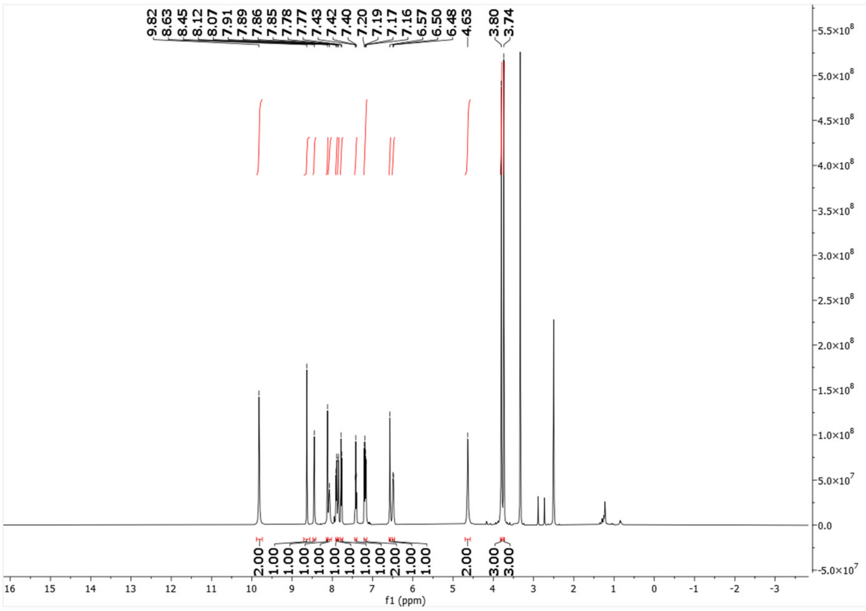

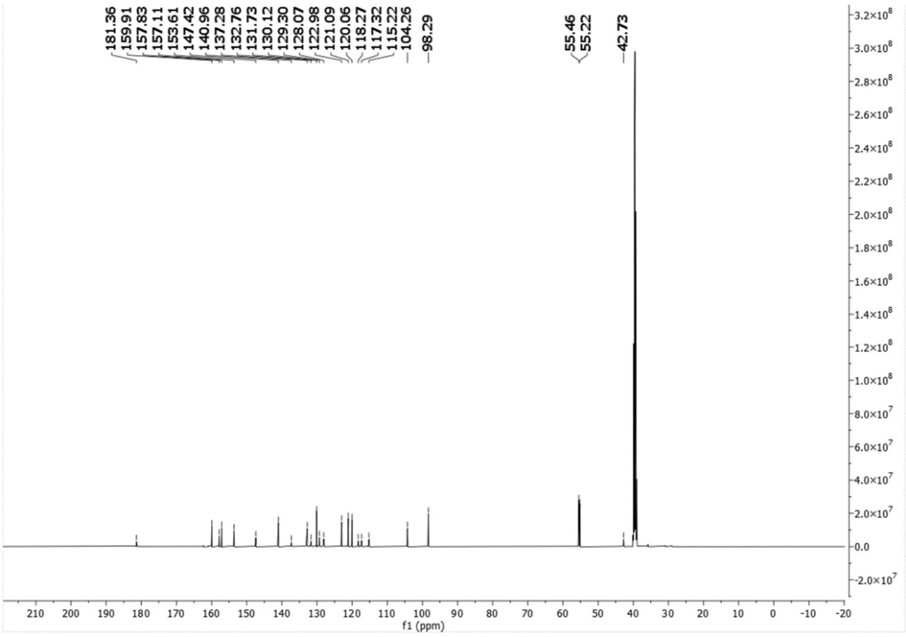

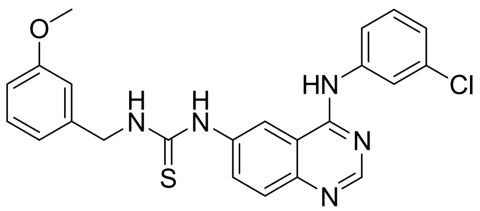
O
H
N
H
N
HN
Cl
N
S
N

8

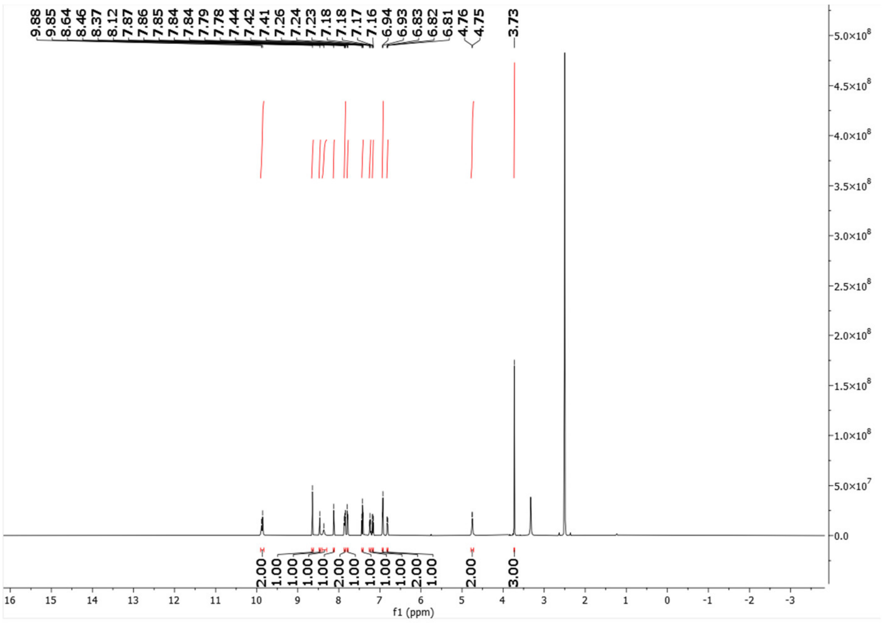
9.88 9.85 8.64 8.46 8.37 8.12 7.87 7.86 7.85 7.84 7.84 7.79 7.78 7.44 7.42 7.41 7.26 7.24 7.23 7.18 7.18 7.17 7.16 6.94 6.93 6.83 6.82 6.81 4.76 4.75
3.73
2.00 1.00 1.00 1.00 1.00 2.00 1.00 1.00 1.00 1.00 2.00 1.00
2.00
3.00
f1 (ppm)

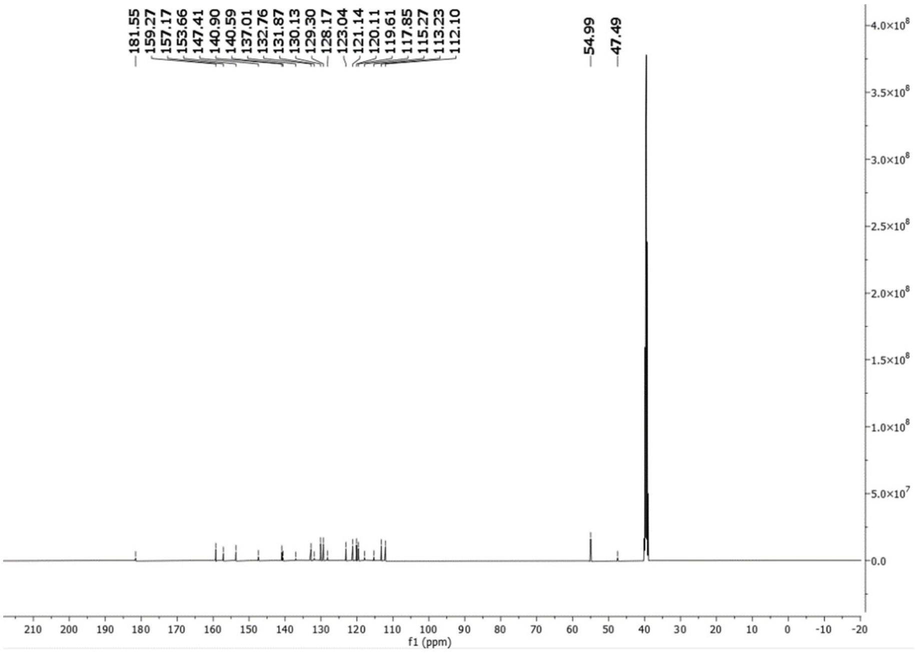
181.55 159.27 157.17 153.66 147.41 140.90 140.59 137.01 132.76 131.87 130.13 129.30 128.17 123.04 121.14 120.11 119.61 117.85 115.27 113.23 112.10
54.99
47.49
f1 (ppm)

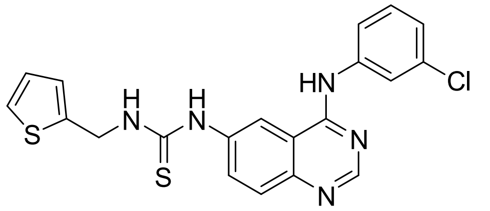

HN
Cl
H
H
N
N
N
S
S
N

9

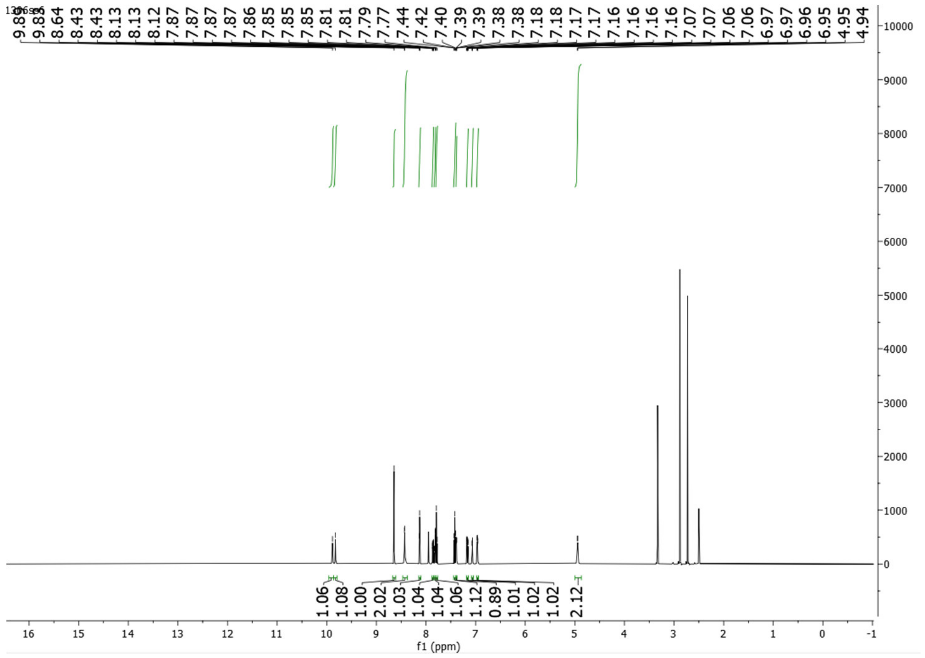

8.64
8.43
8.43
8.13
8.13
8.12
7.87
7.87
7.87
7.87
7.86
7.85
7.85
7.85
7.81
7.81
7.79
7.77
7.44
7.42
7.40
7.39
7.39
7.38
7.38
7.18
7.18
7.17
7.17
7.16
7.16
7.16
7.16
7.07
7.07
7.06
7.06
6.97
6.96
6.95
6.95
4.95
4.94
1.06
1.08
1.00
2.02
1.03
1.04
1.04
1.06
1.12
0.89
1.01
1.02
1.02
2.12
f1 (ppm)

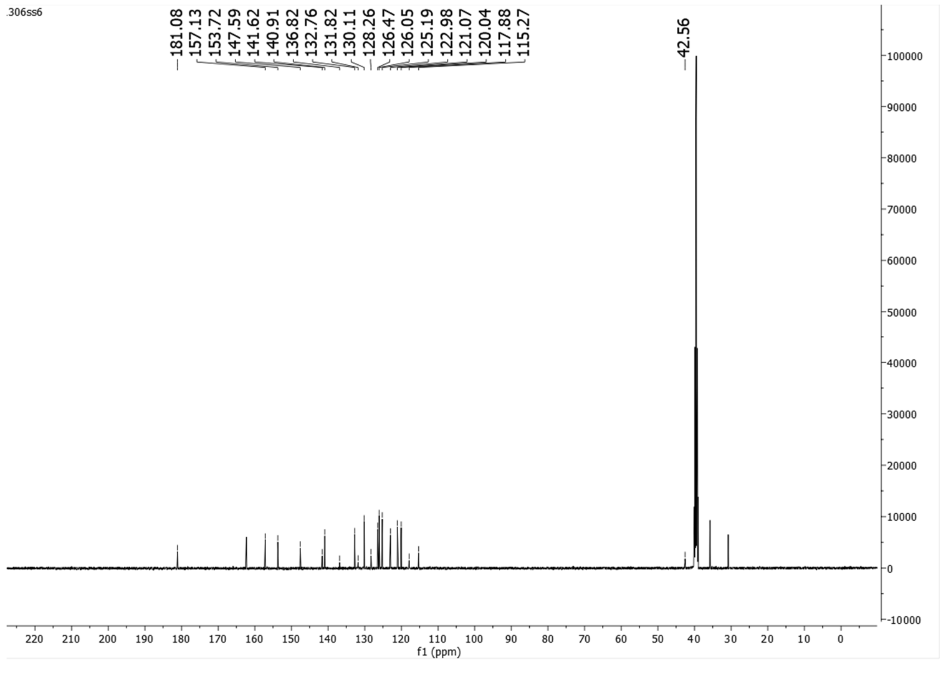

.306ss6
181.08
157.13
153.72
147.59
141.62
140.91
136.82
132.76
131.82
130.11
128.26
126.47
126.05
125.19
122.98
121.07
120.04
117.88
115.27
42.56
f1 (ppm)

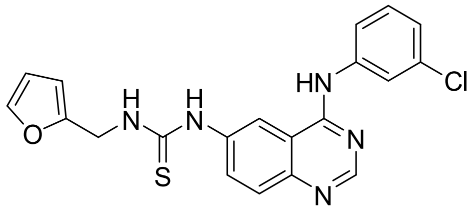

10

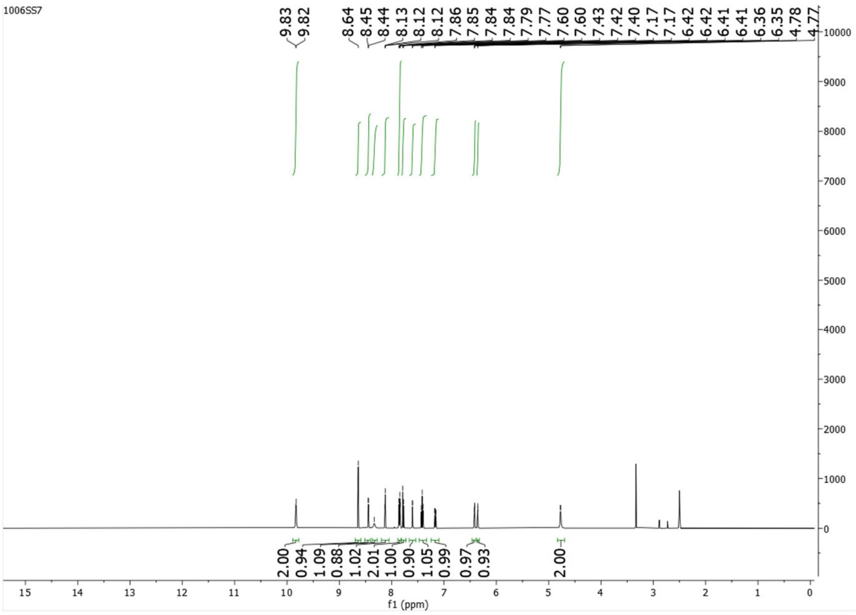

1006SS7
9.83
9.82
8.64
8.45
8.44
8.13
8.12
8.12
7.86
7.85
7.84
7.84
7.79
7.77
7.60
7.60
7.43
7.42
7.40
7.17
7.17
6.42
6.42
6.41
6.41
6.36
6.35
4.78
4.77
2.00
0.94
1.09
0.88
1.02
2.01
1.00
0.90
1.05
0.99
0.97
0.93
2.00
f1 (ppm)

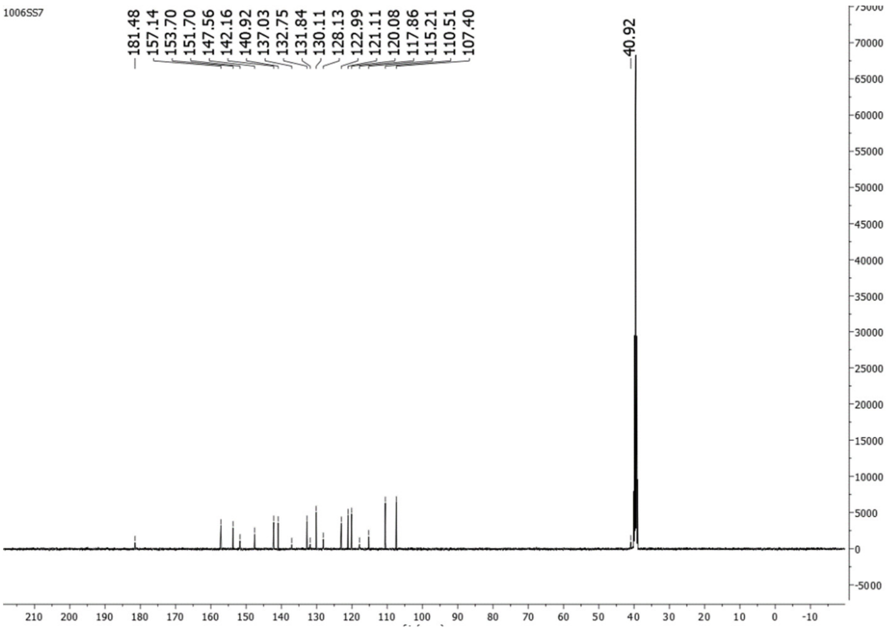

1006SS7
181.48
157.14
153.70
151.70
147.56
142.16
140.92
137.03
132.75
131.84
130.11
128.13
122.99
121.11
120.08
117.86
115.21
110.51
107.40
40.92

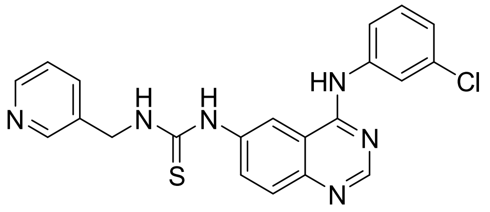
HN
Cl
H
N
H
N
N
N
N
S

11

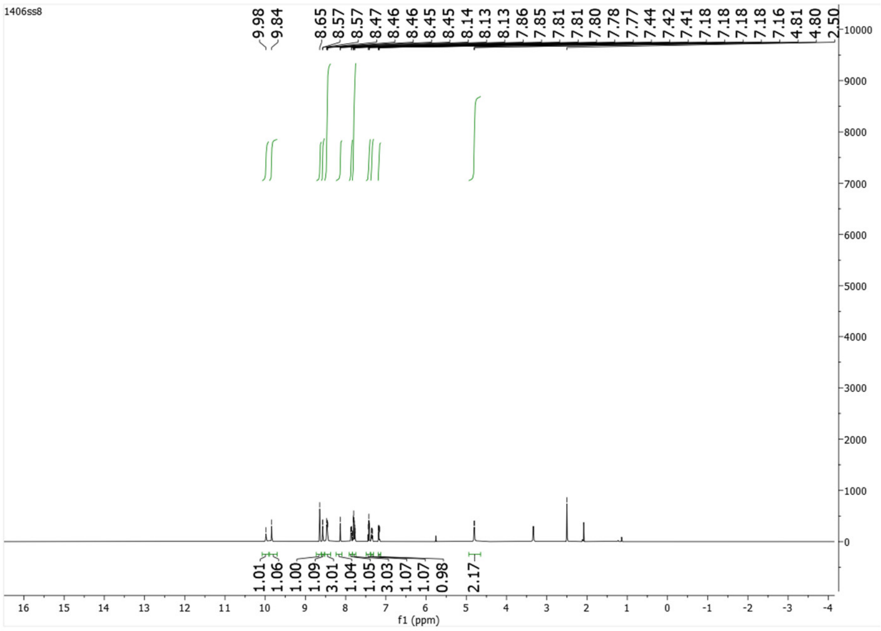
1406ss8
9.98
9.84
8.65
8.57
8.57
8.47
8.46
8.46
8.45
8.45
8.14
8.13
8.13
7.86
7.85
7.81
7.81
7.80
7.78
7.77
7.44
7.42
7.41
7.18
7.18
7.18
7.18
7.16
4.81
4.80
2.50
1.01
1.06
1.00
1.09
3.01
1.04
1.05
3.03
1.07
1.07
0.98
2.17
f1 (ppm)

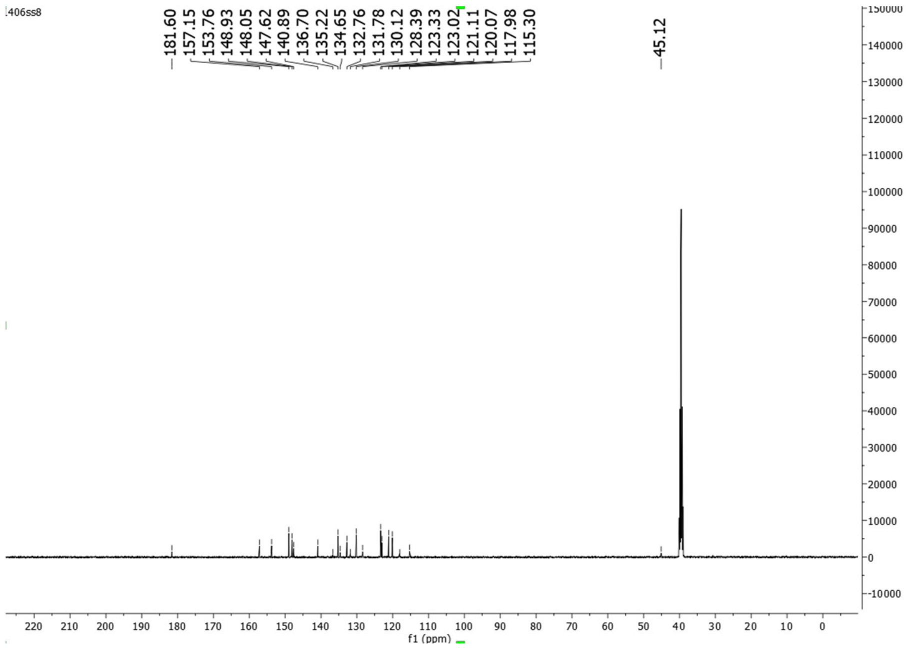
1406ss8
181.60
157.15
153.76
148.93
148.05
147.62
140.89
136.70
135.22
134.65
132.76
131.78
130.12
128.39
123.33
123.02
121.11
120.07
117.98
115.30
45.12
f1 (ppm)

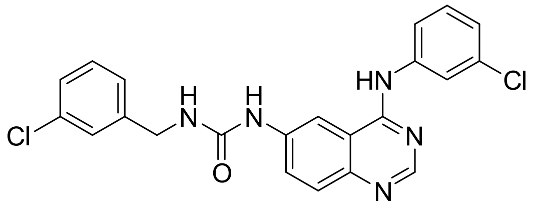

12

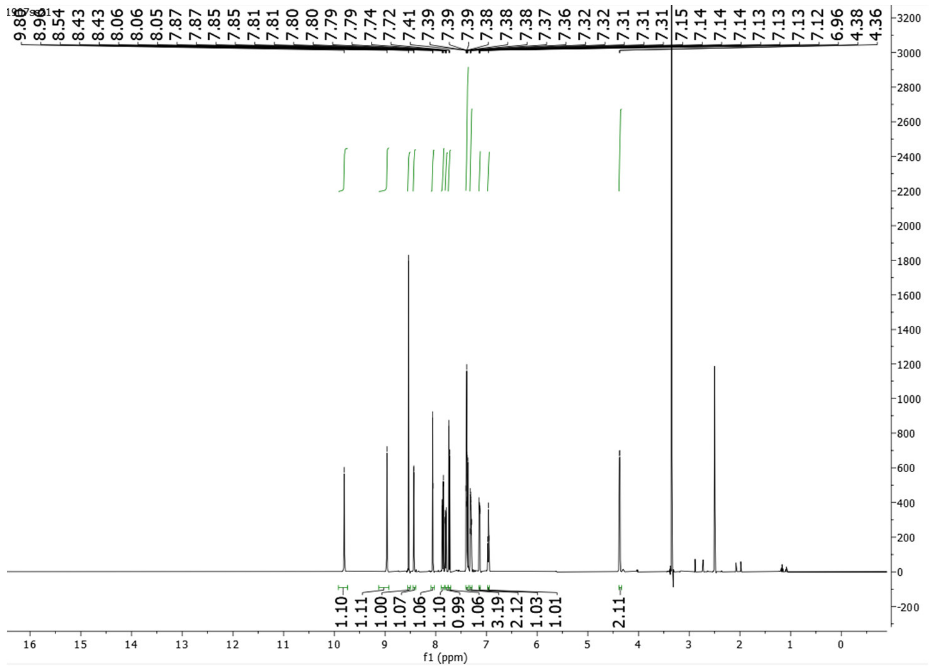

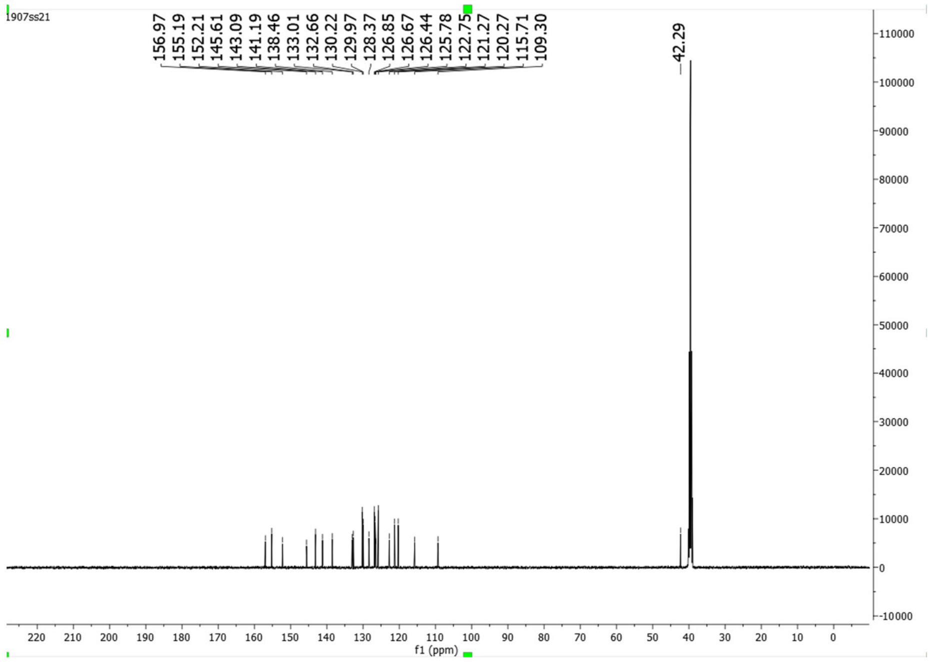

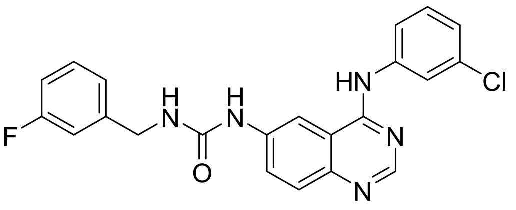
HN
Cl
F
H
N
H
N
O
N
N

13

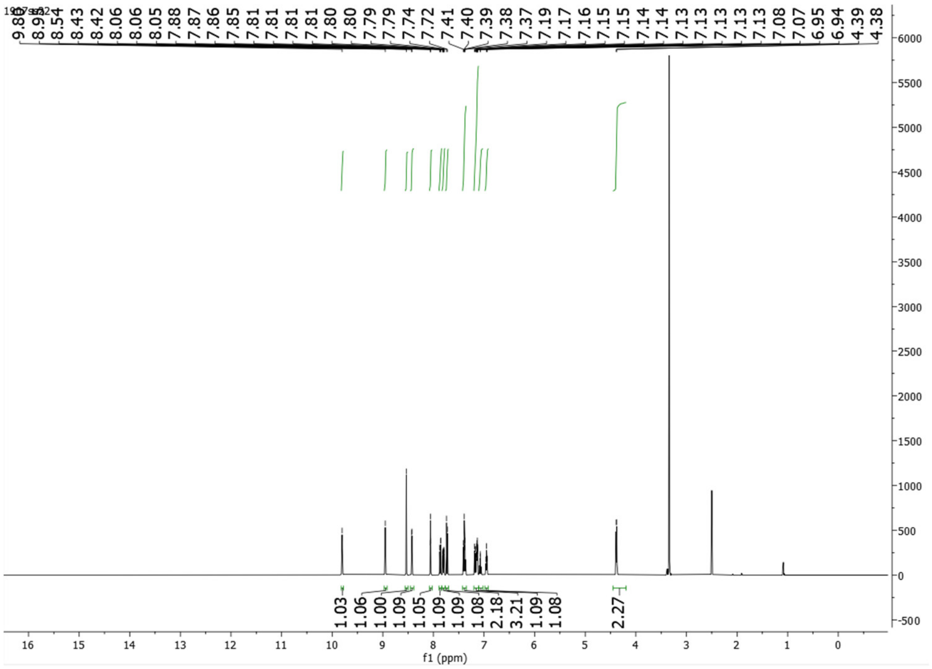
1907ss22
9.80
8.95
8.54
8.43
8.42
8.06
8.06
8.05
7.88
7.87
7.86
7.85
7.81
7.81
7.81
7.81
7.80
7.80
7.79
7.79
7.74
7.72
7.41
7.40
7.39
7.38
7.37
7.19
7.17
7.16
7.15
7.15
7.14
7.13
7.13
7.13
7.13
7.13
7.13
7.08
7.07
6.95
6.94
4.39
4.38
1.03
1.06
1.00
1.09
1.05
1.09
1.09
1.08
2.18
3.21
1.09
1.08
2.27
f1 (ppm)

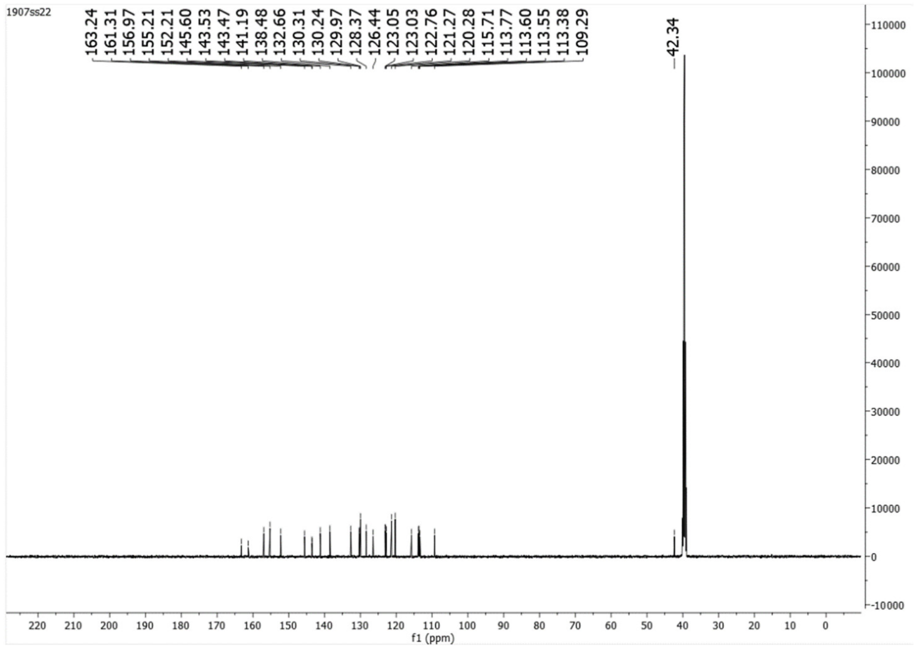
1907ss22
163.24
161.31
156.97
155.21
152.21
145.60
143.53
143.47
141.19
138.48
132.66
130.31
130.24
129.97
128.37
126.44
123.05
123.03
122.76
121.27
120.28
115.77
115.71
113.60
113.55
113.38
109.29
42.34
f1 (ppm)

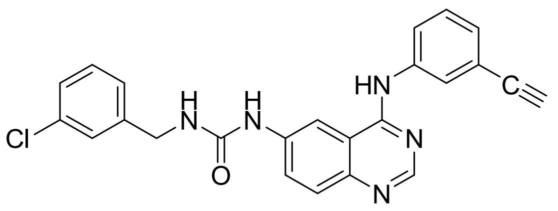

14

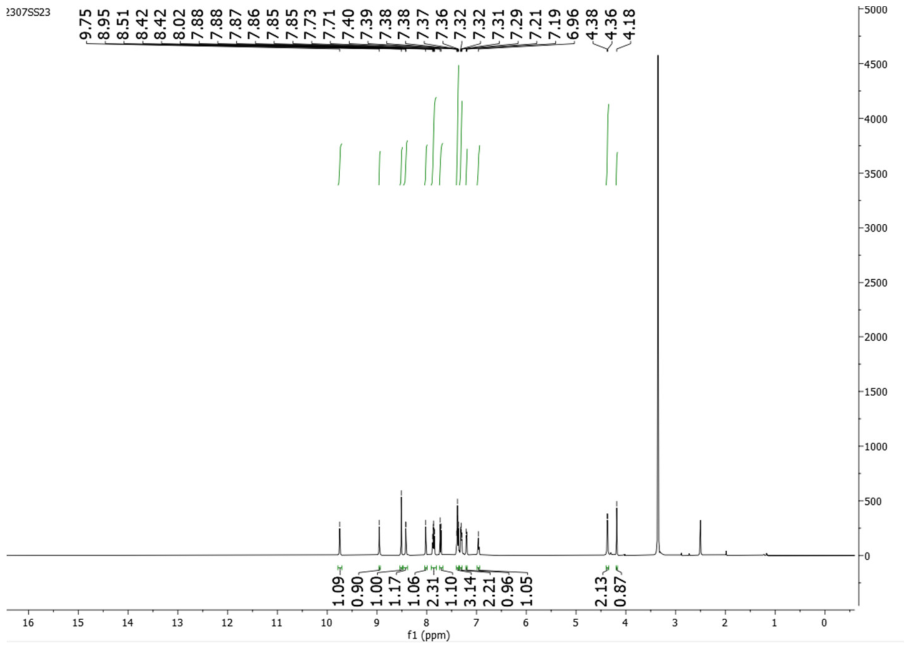

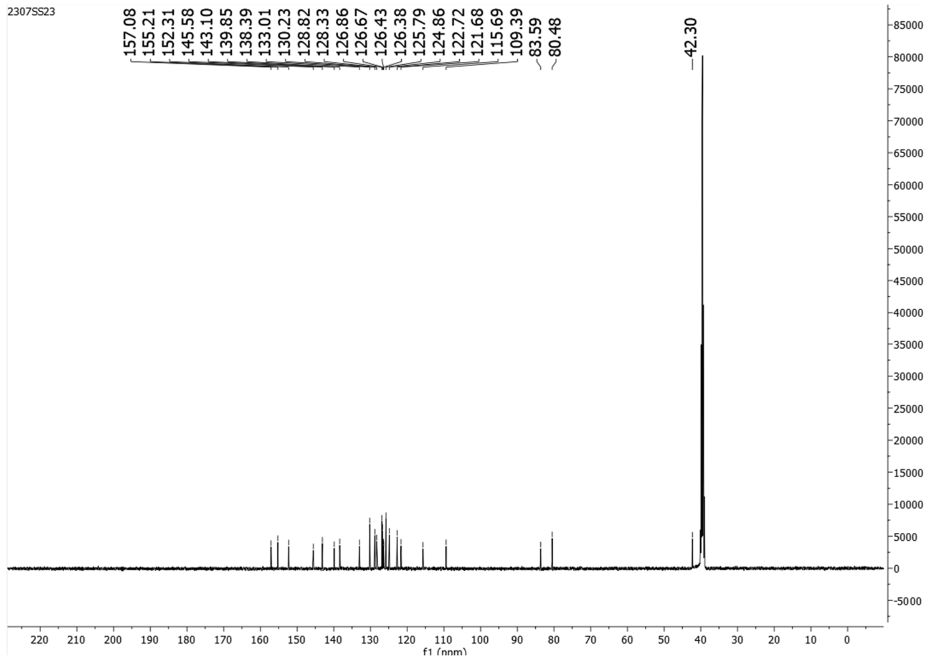

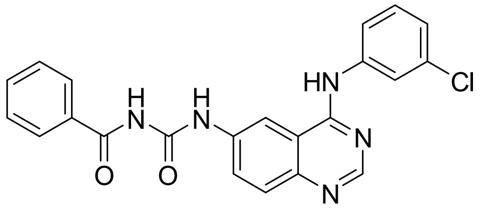
HN
Cl
H
N
H
N
N
N
O
O

15

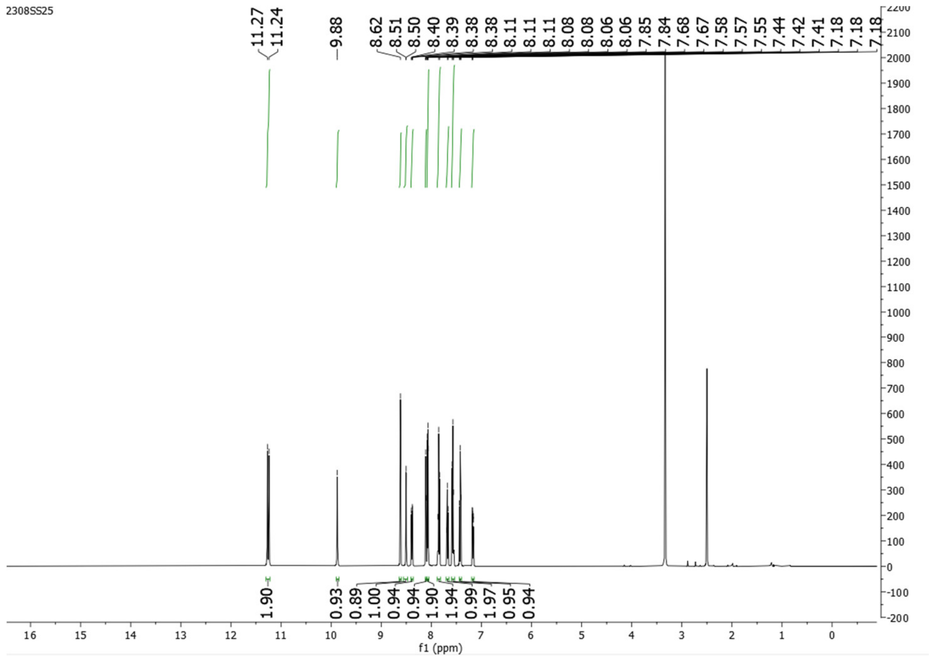
2308SS25
11.27
11.24
9.88
8.62
8.51
8.50
8.40
8.39
8.38
8.38
8.11
8.11
8.11
8.08
8.08
8.06
7.85
7.84
7.68
7.67
7.58
7.57
7.55
7.44
7.42
7.41
7.18
7.18
1.90
0.93
0.89
1.00
0.94
0.94
1.90
1.94
0.99
1.97
0.95
0.94
f1 (ppm)

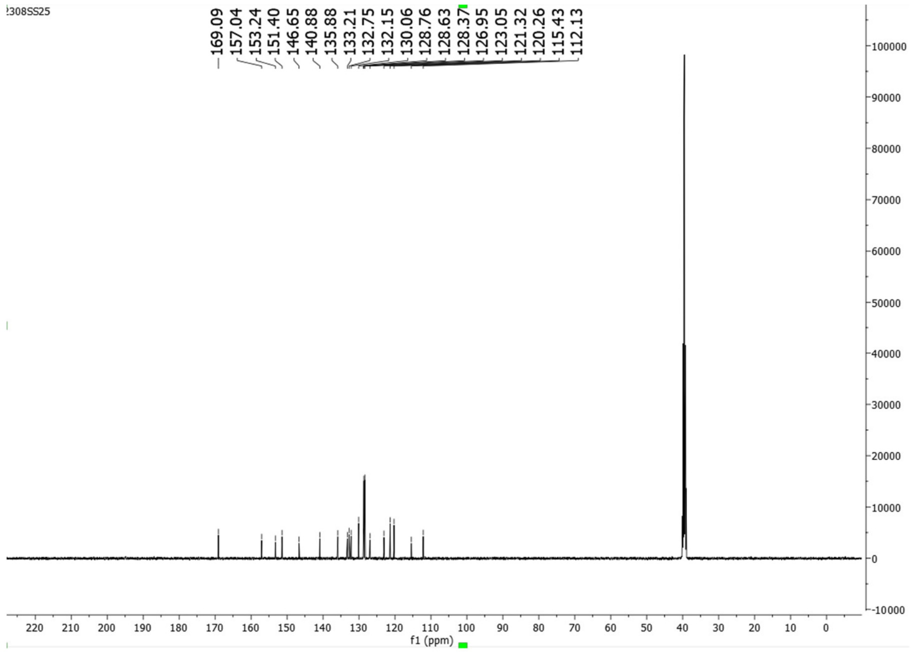
2308SS25
169.09
157.04
153.24
151.40
146.65
140.88
135.88
133.21
132.75
132.15
130.06
128.76
128.63
128.37
126.95
123.05
121.32
120.26
115.43
112.13
f1 (ppm)

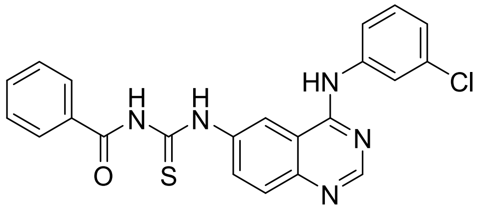

16

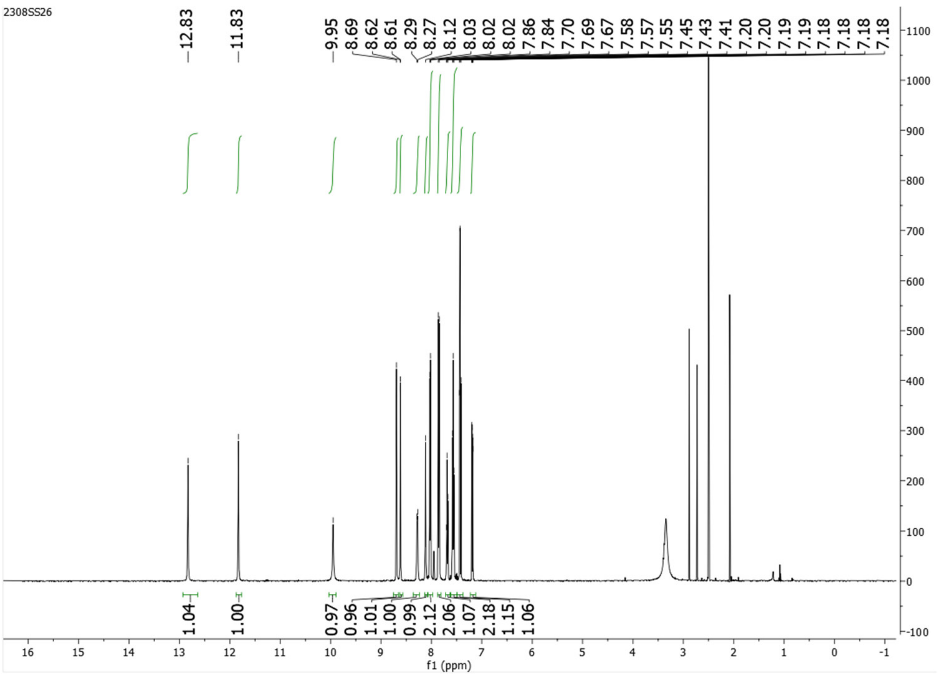
2308SS26
12.83
11.83
9.95
8.69
8.62
8.61
8.29
8.27
8.12
8.03
8.02
8.02
7.86
7.84
7.70
7.69
7.67
7.58
7.57
7.55
7.45
7.43
7.41
7.20
7.19
7.19
7.18
7.18
7.18
7.18
1.04
1.00
0.97
0.96
1.01
1.00
0.99
2.12
2.06
1.07
2.18
1.15
1.06
f1 (ppm)

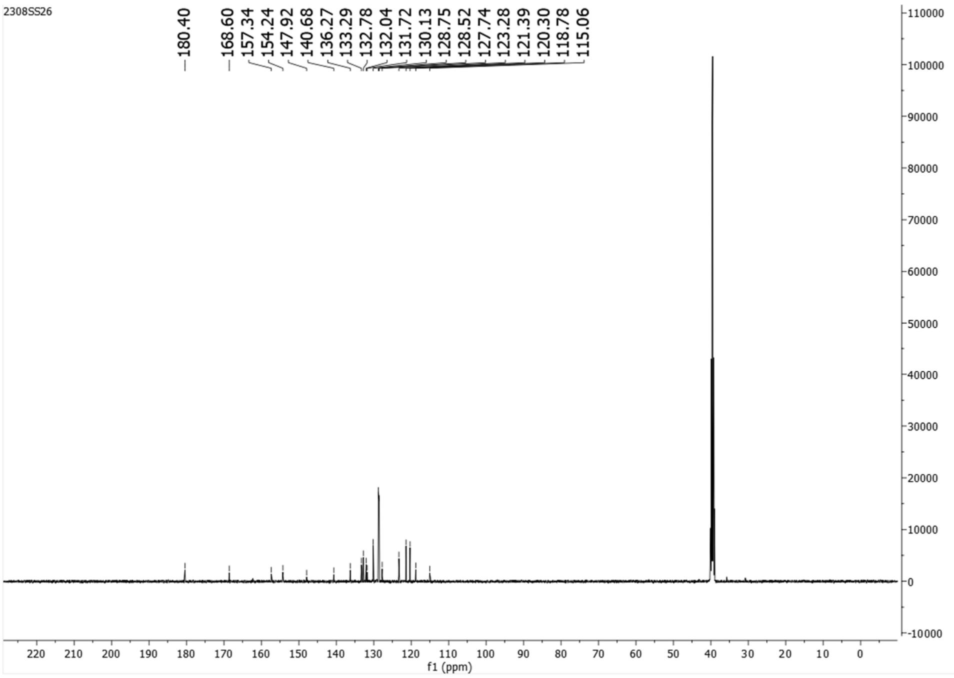
2308SS26
180.40
168.60
157.34
154.24
147.92
140.68
136.27
133.29
132.78
132.04
131.72
130.13
128.75
128.52
127.74
123.28
121.39
120.30
118.78
115.06
f1 (ppm)

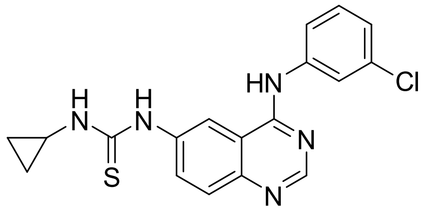

H
N
H
N
HN
Cl
N
N
S

17

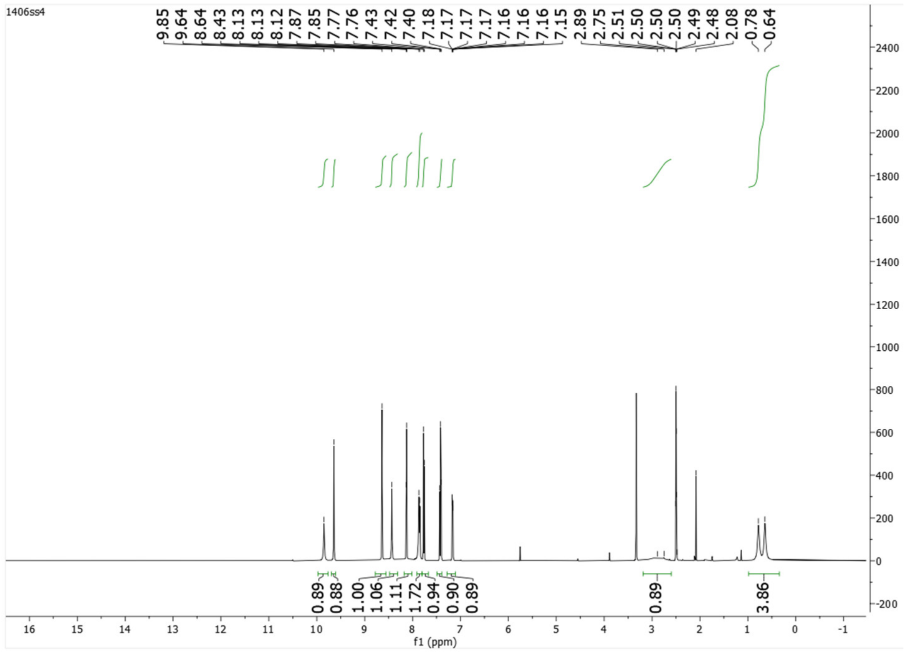

1406ss4
9.85
9.64
8.64
8.43
8.13
8.13
8.12
7.87
7.85
7.77
7.76
7.43
7.42
7.40
7.18
7.17
7.17
7.17
7.16
7.16
7.16
7.15
2.89
2.75
2.51
2.50
2.50
2.50
2.49
2.48
2.08
0.78
0.64
0.89
0.88
1.00
1.06
1.11
1.72
0.94
0.90
0.89
0.89
3.86
f1 (ppm)

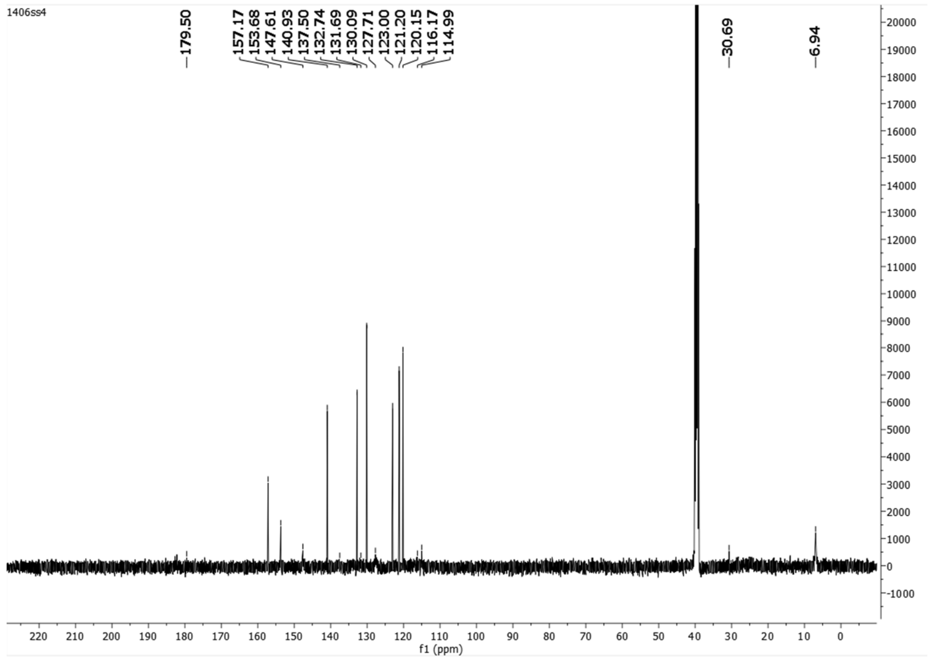

1406ss4
179.50
157.17
153.68
147.61
140.93
137.50
132.74
131.69
130.09
127.71
123.00
121.20
120.15
116.17
114.99
30.69
6.94
f1 (ppm)

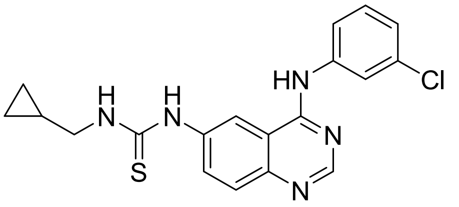
HN
Cl
H
N
H
N
N
S
N

18

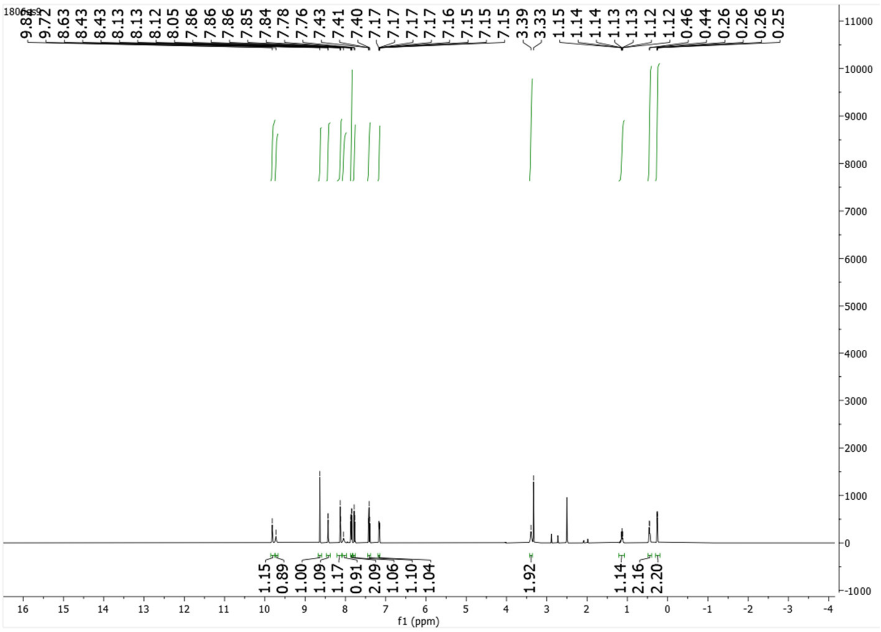
1806ss9
9.83 9.72 8.63 8.43 8.43 8.13 8.13 8.12 8.05 7.86 7.86 7.86 7.85 7.84 7.78 7.76 7.43 7.41 7.40 7.17 7.17 7.17 7.17 7.16 7.15 7.15 7.15 3.39 3.33 1.15 1.14 1.14 1.13 1.13 1.12 1.12 0.46 0.44 0.26 0.26 0.26 0.25
1.15 0.89 1.00 1.09 1.17 0.91 2.09 1.06 1.10 1.04 1.92 1.14 2.16 2.20
f1 (ppm)

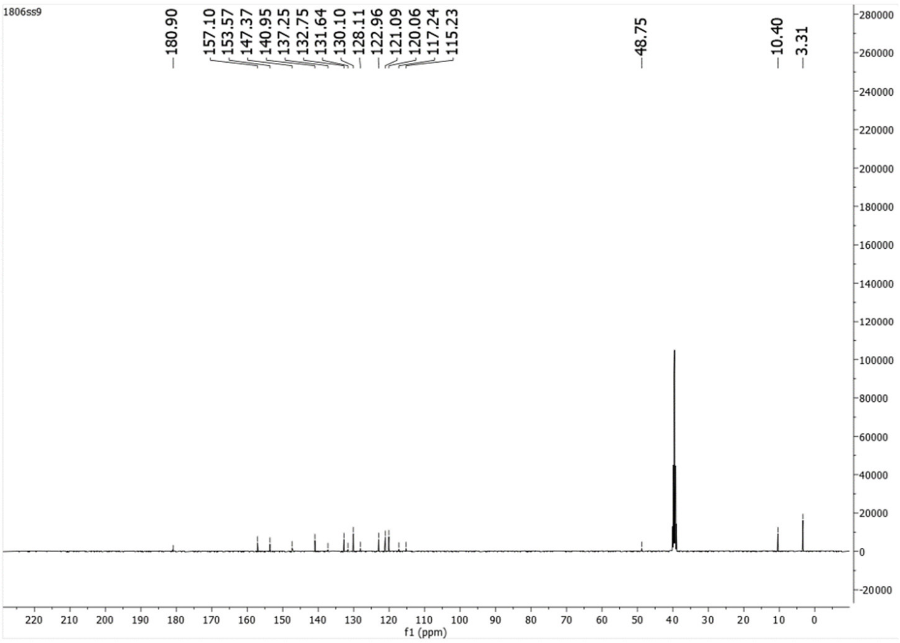
1806ss9
180.90
157.10 153.57 147.37 140.95 137.25 132.75 131.64 130.10 128.11 122.96 121.09 120.06 117.24 115.23
48.75
10.40
3.31
f1 (ppm)

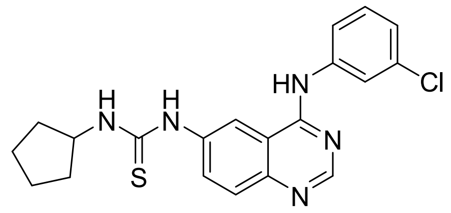
HN
Cl
H
N
H
N
N
S
N

19

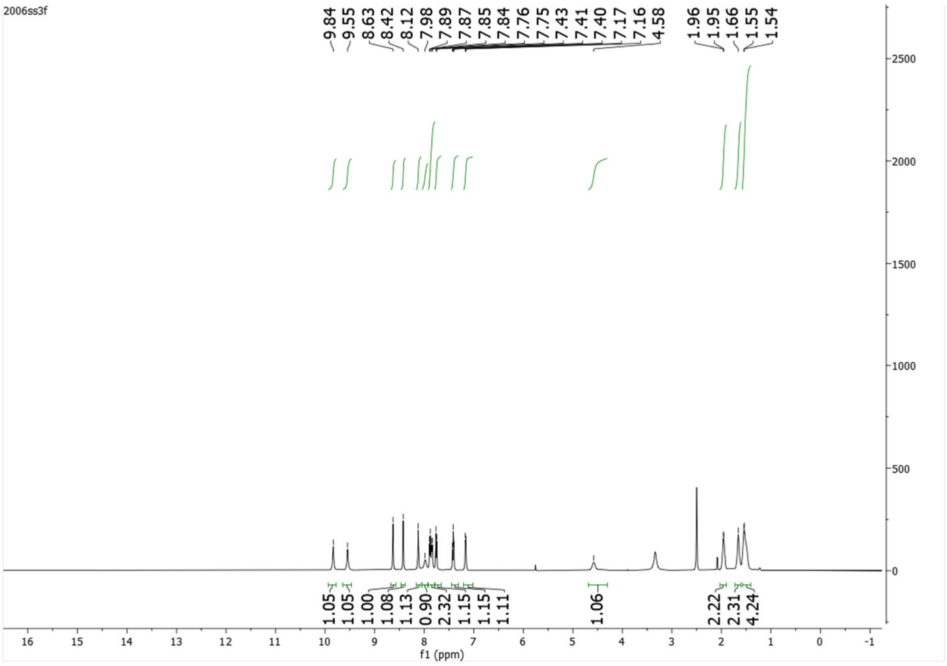
2006ss3f
9.84
9.55
8.63
8.42
8.12
7.98
7.89
7.87
7.85
7.84
7.76
7.75
7.43
7.41
7.40
7.17
7.16
4.58
1.96
1.95
1.66
1.55
1.54
1.05
1.05
1.00
1.08
1.13
0.90
2.32
1.15
1.15
1.11
1.06
2.22
2.31
4.24
f1 (ppm)

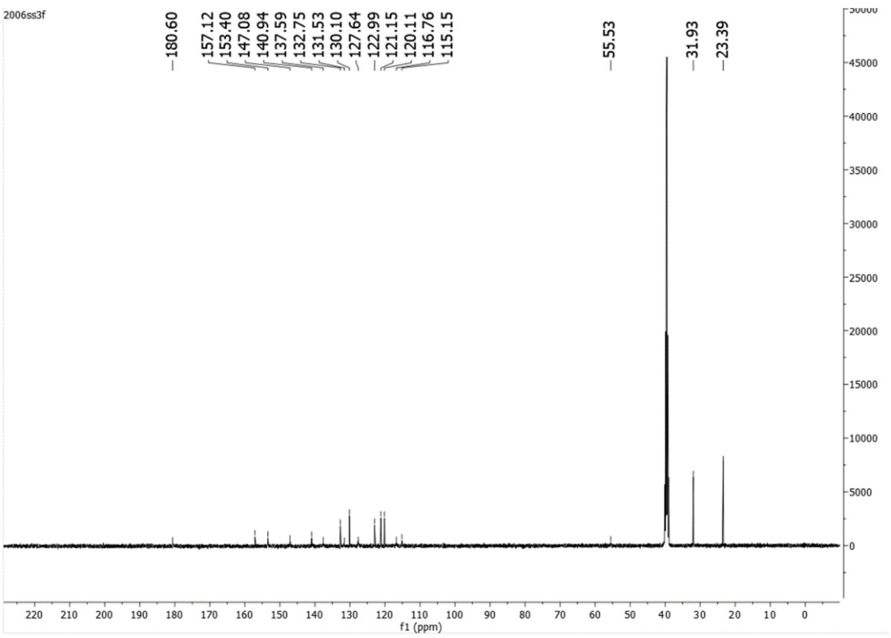
2006ss3f
180.60
157.12
153.40
147.08
140.94
137.59
132.75
131.53
130.10
127.64
122.99
121.15
120.11
116.76
115.15
55.53
31.93
23.39
f1 (ppm)

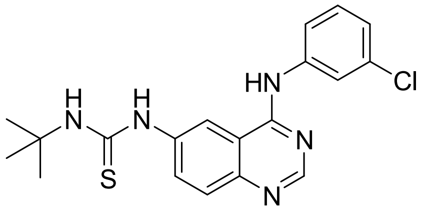
H
N
H
N
S
HN
Cl
N
N

20

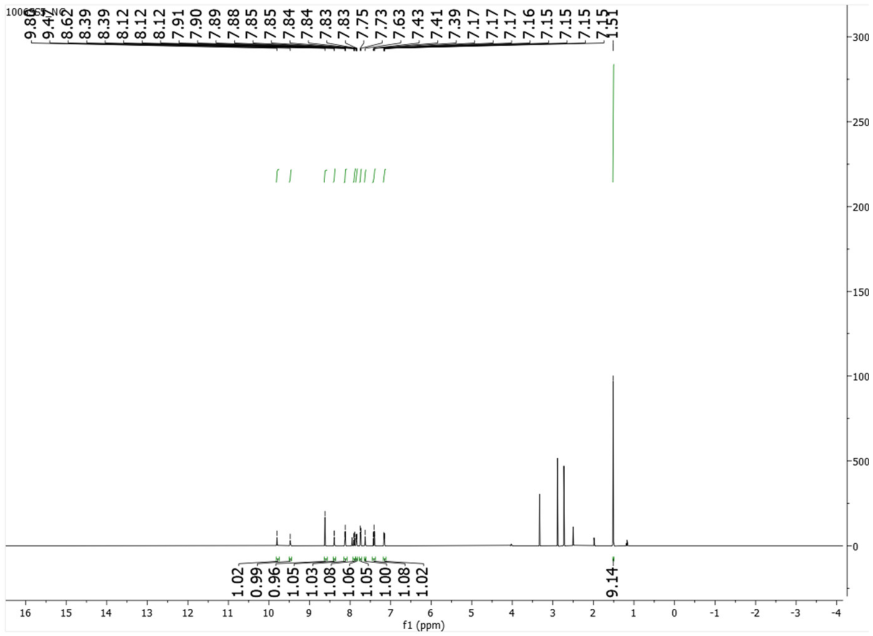
1006SS5_NC
9.80
9.47
8.62
8.39
8.39
8.12
8.12
8.12
7.91
7.90
7.89
7.88
7.85
7.85
7.84
7.84
7.83
7.83
7.75
7.73
7.63
7.43
7.41
7.39
7.17
7.17
7.17
7.16
7.15
7.15
7.15
7.15
1.51
1.02
0.99
0.96
1.05
1.03
1.08
1.06
1.05
1.00
1.08
1.02
9.14
f1 (ppm)

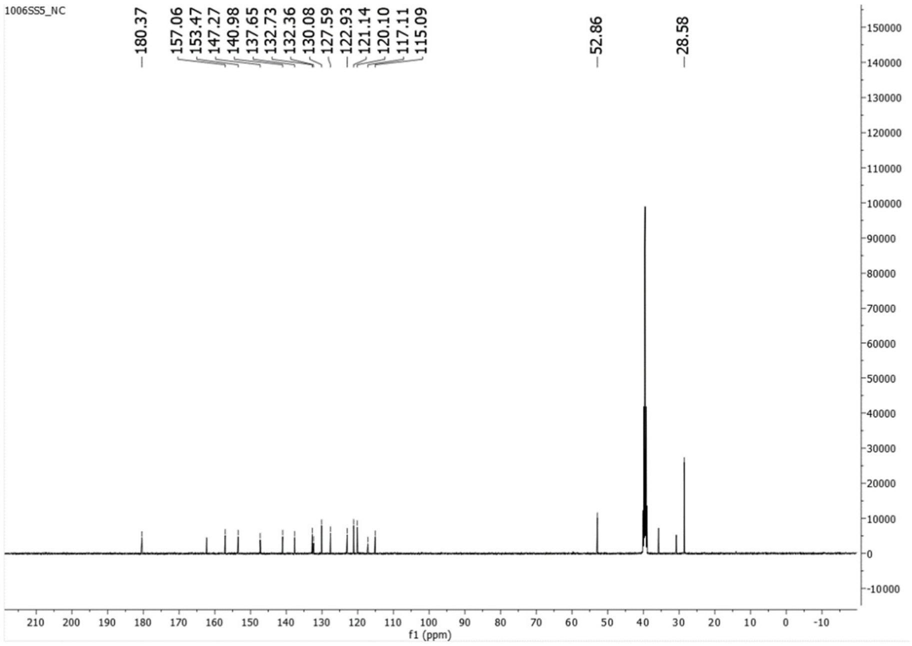
1006SS5_NC
180.37
157.06
153.47
147.27
140.98
137.65
132.73
132.36
130.08
127.59
122.93
121.14
120.10
117.11
115.09
52.86
28.58
f1 (ppm)
